# Metabolite Concentration Changes in Humans After a Bout of Exercise: a Systematic Review of Exercise Metabolomics Studies

**DOI:** 10.1186/s40798-020-0238-4

**Published:** 2020-02-10

**Authors:** Daniela Schranner, Gabi Kastenmüller, Martin Schönfelder, Werner Römisch-Margl, Henning Wackerhage

**Affiliations:** 1 Exercise Biology Group, Department of Sport and Health Sciences, Technische Universität München, Munich, Germany; 2 Institute of Bioinformatics and Systems Biology, Helmholtz Zentrum München, Neuherberg, Germany

**Keywords:** Metabolomics, Biomarker, Exercise, Physiology, Energy metabolism

## Abstract

**Background:**

Exercise changes the concentrations of many metabolites, which are small molecules (< 1.5 kDa) metabolized by the reactions of human metabolism. In recent years, especially mass spectrometry-based metabolomics methods have allowed researchers to measure up to hundreds of metabolites in a single sample in a non-biased fashion. To summarize human exercise metabolomics studies to date, we conducted a systematic review that reports the results of experiments that found metabolite concentrations changes after a bout of human endurance or resistance exercise.

**Methods:**

We carried out a systematic review following PRISMA guidelines and searched for human metabolomics studies that report metabolite concentrations before and within 24 h after endurance or resistance exercise in blood, urine, or sweat. We then displayed metabolites that significantly changed their concentration in at least two experiments.

**Results:**

Twenty-seven studies and 57 experiments matched our search criteria and were analyzed. Within these studies, 196 metabolites changed their concentration significantly within 24 h after exercise in at least two experiments. Human biofluids contain mainly unphosphorylated metabolites as the phosphorylation of metabolites such as ATP, glycolytic intermediates, or nucleotides traps these metabolites within cells. Lactate, pyruvate, TCA cycle intermediates, fatty acids, acylcarnitines, and ketone bodies all typically increase after exercise, whereas bile acids decrease. In contrast, the concentrations of proteinogenic and non-proteinogenic amino acids change in different directions.

**Conclusion:**

Across different exercise modes and in different subjects, exercise often consistently changes the average concentrations of metabolites that belong to energy metabolism and other branches of metabolism. This dataset is a useful resource for those that wish to study human exercise metabolism.

## Key Points

- This study identified 196 metabolites that significantly change their concentration from pre to 24 h post endurance or resistance exercise in human blood, urine, or sweat in at least two metabolomics experiments.
- A bout of acute exercise typically increases the concentrations of lactate, pyruvate, fatty acids, acylcarnitines, ketone bodies, nucleotides; lowers the concentrations of bile acids; and has mixed effects on proteinogenic and non-proteinogenic amino acids.

## Background

Living organisms are stable systems even though the molecules within organisms constantly change in a myriad of chemical reactions. Based on the Greek word “*metabole*” (English: change), “*metabolism*” is used to describe all the chemical reactions that change molecules in living organisms. Key metabolic discoveries by the early biochemists are the discovery and characterization of glycolysis by Pasteur, Embden, Meyerhof, and Parnas; the discovery of enzymes; the mapping of metabolic pathways; and discoveries linked to biochemical genetics [1, 2]. Our current knowledge of human metabolism is summarized in genome-scale metabolic reconstructions such as the Virtual Metabolic Human database, containing 17730 reactions and 5180 metabolites [3]. Metabolites are the molecules that change or react in metabolic reactions of a living being. Metabolites typically have a molecular mass of less than 1.5 kDa. In addition to our own, endogenous metabolites, metabolic databases also include exogenous metabolites that are produced by microorganisms, residing for example in our intestines, or that are derived from nutrients or drugs, which are termed xenometabolites [4].

### How Does Exercise Affect Metabolism?

While each meal feeds our metabolic pathways with new metabolites, nothing quite changes the rates of metabolic reactions as much as a bout of intensive exercise [5]. On a whole-body level, oxygen uptake rises from 0.25 l/min at rest to 5 l/min during maximal exercise in a trained athlete. This is an energy turnover of ≈5 kJ/min at rest which equates to 0.3 g of glucose per minute to ≈100 kJ/min during maximal exercise which equates to 6 g of glucose per minute. The fact that there is only a total of ≈4 g of free glucose in a human being [6] demonstrates the challenge that such a rise of energy expenditure poses to the metabolism of the exercising individual.

The fold-changes of metabolic reactions in the working muscles are even greater. When changing from rest to exercise, the rate of adenosine triphosphate (ATP) hydrolysis especially by the force-generating myosin heads of a muscle fiber can increase by more than 100-fold [7]. Given that there are only ≈10 mM of ATP in a muscle fiber [8] and given that a major drop of the ATP concentration will cause *rigor mortis*, ATP-synthesizing reactions must immediately increase their rate so that ATP re-synthesis matches ATP hydrolysis within fractions of a second [9].

Exercise also affects hormone concentrations which is relevant as many hormones are technically classified as metabolites. Here, the best characterized exercise change is the increase of catecholamines [10, 11] that helps to increase heart rate and cardiac contractility as well as adjusts metabolism and blood flow. Finally, resistance exercise not only increases muscle protein synthesis for several days post exercise [12] but also elevates muscle protein breakdown [13]. While proteins are not classified as metabolites, the amino acids that constitute them are metabolites and either ingested or synthesized in metabolic reactions. Collectively, this demonstrates that the three major branches of metabolism, which are energy metabolism, anabolism, and catabolism, are profoundly changed in response to a bout of exercise.

### How Are Metabolites Measured and What Is Metabolomics?

The concentrations of metabolites in biofluids such as blood, urine, and saliva have traditionally been measured one-by-one with enzyme assays followed by fluorometric or spectrophotometric detection [14]. This, however, has changed with the advent of metabolomics methods. Metabolomics describes methods that allow the high throughput quantification of hundreds of metabolites in a single sample. This is mostly achieved through the separation of metabolites via liquid or gas chromatography followed by the detection of individual metabolites through their specific mass-to-charge ratio (m/z) and their induced break-down (fragmentation) in a mass spectrometer. The retention time from the chromatographic separation, the mass-to-charge ratio, and the fragmentation pattern are characteristic features for each ionized metabolite. This information can therefore be used to identify the detected metabolites through matching against databases of known metabolites [4]. While nuclear magnetic resonance spectroscopy-based metabolomics methods are also available and have specific advantages [15], mass spectrometry-based metabolomics methods dominate. Further variations of metabolomics are untargeted or global metabolomics which measures all detectable metabolites in a sample versus targeted metabolomics where a specific subset of metabolites is measured [4]. Collectively, the improvements in metabolomics methods allow researchers to detect more and more metabolites in human body fluids [16].

While exercise physiologists have traditionally focused on measuring individual metabolites such as lactate [17], they have since 2009 used metabolomics methods to obtain a global view of how exercise changes metabolite concentrations [18]. The plethora of different metabolites and methodologies used in these studies makes it difficult to obtain a comprehensive overview over how a bout of exercise changes human metabolite concentrations in different body fluids and organs.

The aim of this project was therefore to conduct a systematic literature analysis to review all published studies where researchers used mass spectrometry or nuclear magnetic spectroscopy-based metabolomics to study the effect of exercise on metabolite concentrations. Specifically, we report and discuss metabolites that significantly change their concentration in mass spectrometry or nuclear magnetic resonance-based human metabolomics studies after a single bout of exercise. In our analysis, we found 196 metabolites that significantly change their concentration within 24 h after a bout of exercise in at least two studies within human blood or other body fluids.

## Methods

### Search Strategy

**Fig. 1:**
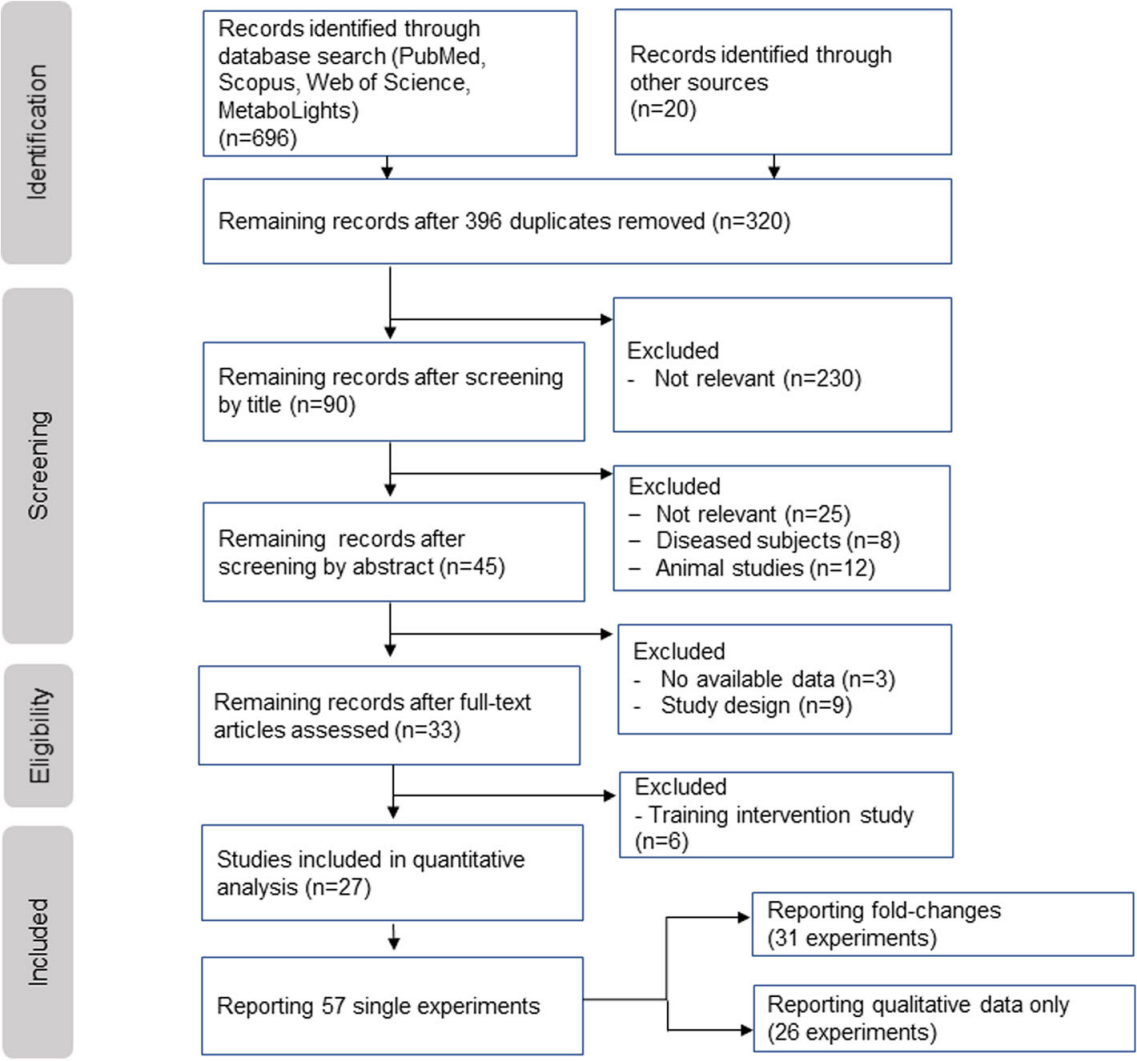
PRISMA flowchart from the systematic literature search

To identify publications that use a metabolomics approach to measure metabolite changes after a bout of exercise, we carried out a systematic review following the PRISMA guidelines [19]. We searched four different literature databases using the PICO (Population, Intervention, Comparison, Outcome) strategy [20]. This search strategy combines the parameters of the research question into one search string to find relevant studies (Fig. 1).

For the literature search, we only used the parameters “*intervention*” and “*comparison*” of the PICO search strategy in the search terms because the eligible studies did not need to have a control group. To get a first overview, we searched PubMed using the search term (“*metabolomics*” AND “*exercise*”) and repeated this search in PubReMiner to extract additional, relevant search terms. We ended up with the search terms ((“*metabolomics*” [*MeSH Terms*] *OR* “*metabolome*” [*MeSH Terms*]) *OR sportomics* [*All Fields*]) *AND* “*exercise*” [*MeSH Terms*]. With these search terms, we conducted two main searches on the third of August 2017 and on the first of June 2018 and searched PubMed (101 abstracts), Web of Science (194 abstracts), Scopus (313 abstracts), and MetaboLights database (88 abstracts). The earliest study matching our search criteria was published in 2010. We included studies from that date onwards.

From the abstracts retrieved, we included articles from peer-reviewed journals, written in English that investigated metabolic changes in humans in response to a bout of exercise. Our eligibility criteria were as follows:

- Adult participants (> 18 years of age) without metabolic disease, malfunction or genetic disorder of metabolism (e.g., diabetes mellitus) and normal weight (BMI > 18 and < 28 kg/m^2^)
- Metabolomics analysis technique based on either mass spectrometry or ^1^H-nuclear magnetic resonance (^1^H-NMR) applied to analyze metabolic changes
- Physical exercise of any kind had to be the primary intervention of the study
- Studies or experiments investigating changes of metabolite concentrations within 24 h after a bout of exercise
- Changes of metabolites had to be significant (raw *p* values of *p* < 0.05) and reported with respect to a resting value before exercise in fold-change, % change or as “decrease” or “increase.” In cases with no fold-change values given, we contacted the corresponding author of the publication to request quantitative data
- Sampling specimen included in the review are serum, plasma, capillary blood, urine, saliva, and sweat

Studies with one or more of the following criteria were excluded:

- Article type: conference proceedings, reviews, comments or letters to editor
- Subjects: animal studies, studies on chronically or acutely ill subjects; studies on overweight, obese, individuals or individuals with eating disorders; studies on children
- Methods: studies where exercise was not the primary intervention and studies that used other methods than mass spectrometry or nuclear magnetic resonance metabolomics methods to measure metabolites (e.g., studies that used biochemical analyzer kits were excluded)
- Outcomes: metabolites within a study that showed no significant change after an exercise intervention
- Studies reporting changes of metabolite concentrations later than 24 h after a bout of exercise or after an exercise training intervention

From every study, the following information was extracted if available: Author, year of publication, subjects (numbers, age, sex, BMI or body fat percentage, training status or cardiorespiratory fitness (VO_2max_), training load per week, intervention of the study (intervention sessions, intensity (%VO_2max_), duration, kind of exercise, nutrition during intervention), samples for metabolomics analysis (number of samples, tissue sampled, nutritional protocol before sampling, interval between exercise and post-exercise sample, fasting/no fasting before sampling, outcomes (number of metabolites detected, quantitative change of significantly altered metabolites, analyses method, database comparison)), and remarks.

### Data Analysis

Significant changes of metabolite concentrations were noted in a table and sorted according to the following metabolite subgroups and their underlying metabolism: carbohydrates and tricarboxylic acid (TCA) cycle intermediates; lipids; amino acids, their derivates and peptides; nucleotides; cofactors/vitamins, and xenometabolites (i.e., non-human metabolites such as drugs or food dyes).

## Results

After removing duplicates and applying exclusion criteria, we read 45 articles full-text of which 33 matched our eligibility criteria. Within these 33 publications, we further excluded six studies that were either exercise training studies or reported data that were not measured within the 24 h after a single bout of exercise. Within the remaining 27 publications [21–49], 57 single experiments were reported. Because only six out of these 57 experiments used resistance exercise as an intervention and 51 used endurance exercise, data are presented together but labeled separately.

Out of the 57 experiments, 26 experiments reported significant (*p* < 0.05) increase or decrease of metabolite concentrations qualitatively within 24 h after a bout of exercise without providing the corresponding fold changes. Thirty-one experiments were quantitative, reporting fold-change values for significantly (*p* < 0.05) changed metabolites in blood, urine, saliva, and sweat. The Additional file 1: Tables S1-5 summarize the results from all 57 experiments.

### Subjects

Of 57 experiments, 45 investigated only male subjects (*n* = 307), ten investigated female and male subjects (*n* = 211) and two investigated only female subjects (*n* = 22) which is a ≈10-to-1 male bias that should be rectified in the future. In 23 of the 57 experiments, well-trained athletes were used as subjects. The remaining 24 experiments investigated heterogeneous groups ranging from sedentary to recreationally active subjects. Further details are in Additional file 2: Table S6.

### Exercise Interventions

Fifty-one out of 57 experiments chose endurance exercise such as cycling or running as an exercise intervention in a controlled laboratory or outdoor setting. Exercise duration ranged from 30 min to 96 h. Exercise intensities ranged from moderate (< 60% of VO_2max_) to supramaximal (> 110–300% of the workload achieved at VO_2max_) intensity. In 17 experiments, participants exercised at an intensity around the individual anaerobic threshold (corresponding to ≈60–80% VO_2max_). In a further 13 experiments, participants did self-paced exercise, without a measurement of VO_2_-uptake. Six experiments chose resistance exercise such as leg press as an exercise intervention on male subjects only. For details, see Additional file 2: Table S6.

### Sample Type and Timing

**Fig. 2:**
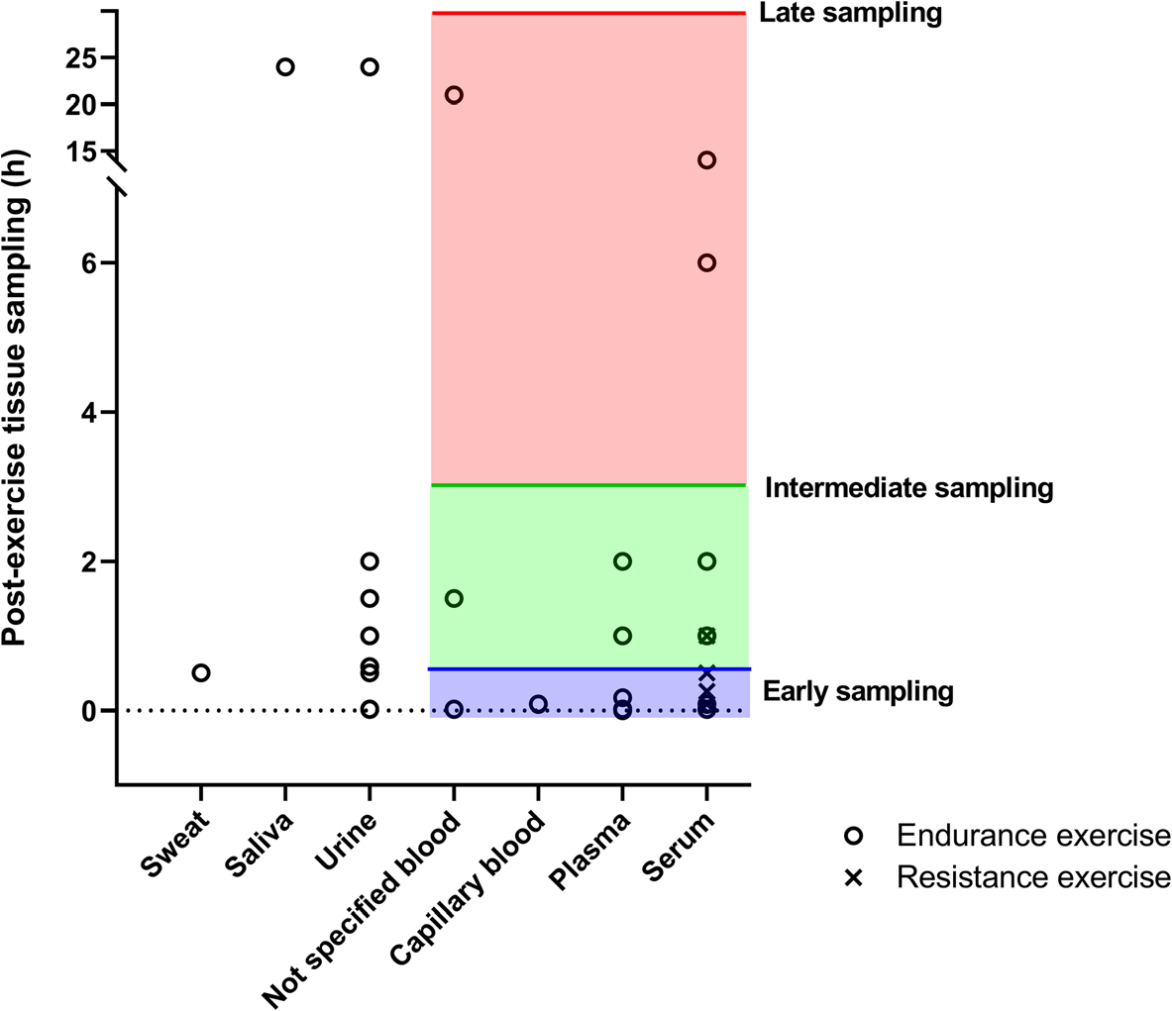
Distribution of post-exercise sample type and timing across all 57 experiments. Note that only experiments with blood samples are color-coded and categorized into three timing categories. Blue: early samples; green: intermediate samples; red: late samples

Forty of 57 experiments used human blood (plasma, serum, capillary, or non-specified blood), thirteen used urine, three saliva, and one experiment sweat to determine metabolite concentration changes after exercise (Fig. 2).

With respect to timing, each included study compared one or more post-exercise samples with a baseline or pre-exercise sample. The earliest post-exercise samples across all tissue types were drawn immediately after exercise, the latest 24 h after exercise. Due to this heterogeneity in sample timing, we categorized all experiments with human blood samples into three categories: early (0–0.5 h after exercise), intermediate (> 0.5–3 h after exercise), and late (> 3–24 h after exercise) (Fig. 2).

### Metabolites With Significant Concentration Changes After a Bout of Exercise

**Fig. 3:**
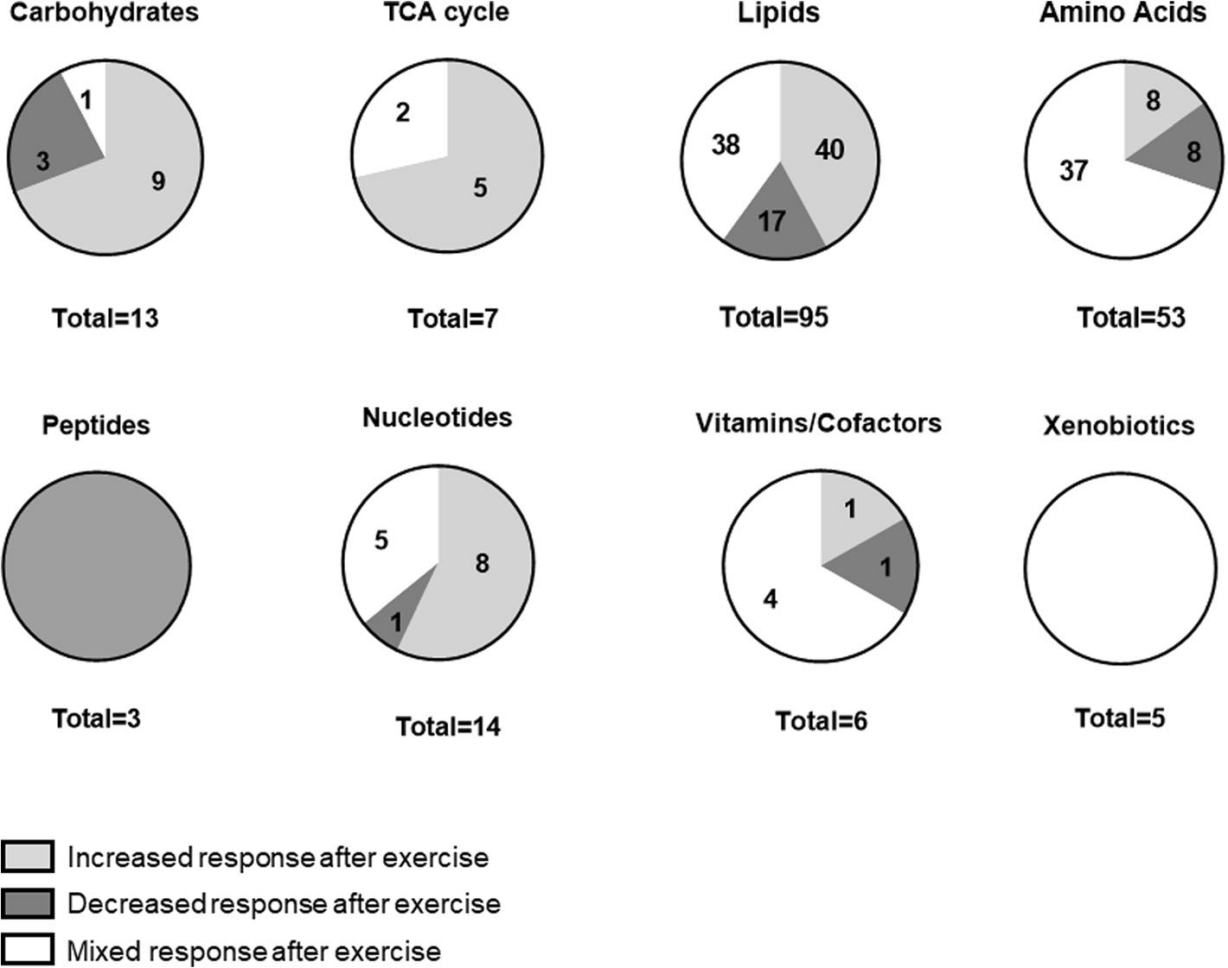
Responses of each metabolite class within 24 h after a bout of exercise colored by direction of effects

In total, 196 metabolites changed significantly in at least two out of the 57 experiments. We used this requirement to limit the number of metabolites that we report to a manageable level and to increase reliability. The 196 exercise-responsive metabolites belong to different metabolite classes. They include 13 carbohydrates, 95 lipids, seven tricarboxylic acid (TCA or Krebs) cycle metabolites, 53 amino acids and their derivatives, three peptides, 14 nucleotides, six vitamins and cofactors, and five xenobiotics. Out of 196, 106 metabolites changed in the same direction after exercise in all experiments: 71 metabolites were solely reported to increase, 35 solely to decrease. Ninety metabolites of 196 showed mixed responses between experiments (Fig. 3). Among these 90 mixed cases, 38 metabolites were determined in the same biofluid such as blood plasma. Twenty-two of the 90 mixed responses were sampled in different blood samples like serum, plasma, or capillary blood. Within the metabolite subgroups, amino acids were those that accounted for most of the inconsistent findings. Within 37 mixed cases of amino acid responses after exercise, 19 were measured in the identical biofluid.

**Fig. 4:**
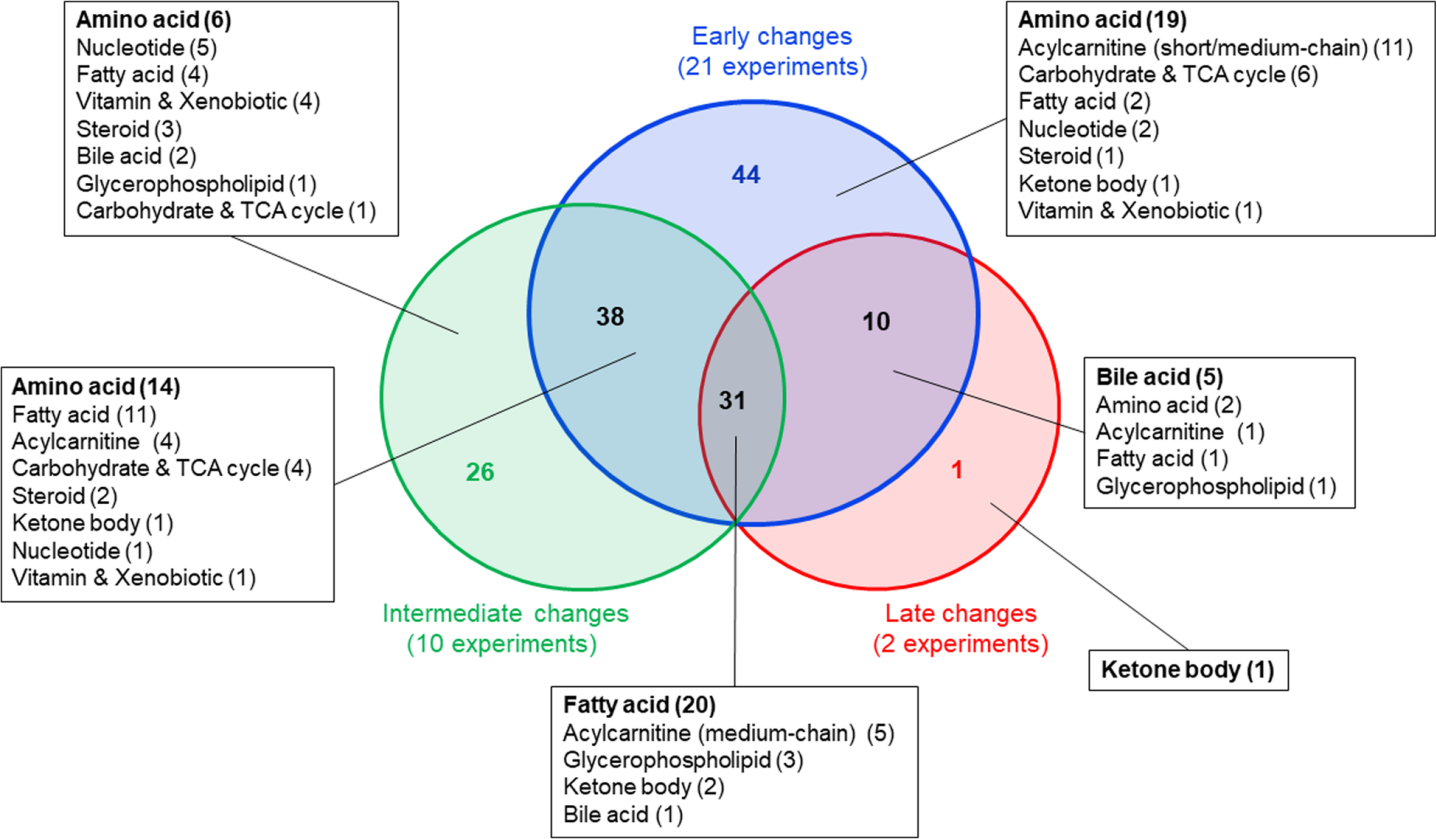
Venn diagram showing the number of metabolites that are changed in relation to time point of sampling. Early changes: within 0.5 h after exercise; Intermediate changes: between > 0.5 and 3 h after exercise; Late changes: between > 3 and 24 h after exercise. The respective metabolites in each sample category (early to late) and the overlapping metabolites are shown in Additional file 3: Table S7. No metabolites were changed equally in intermediate and late only. For example, we found 31 metabolites that are changed at all sampling time points following exercise consisting of acylcarnitines and free fatty acids. Note that only blood metabolite changes of 33 experiments are shown in this diagram.

To identify if metabolite or metabolite class changes vary with post-exercise sample timing, we compared the results for all metabolites detected in blood samples (33 experiments) across the three timing categories (Fig. 4). Though not all metabolites were detected in each experiment and only two experiments were categorized as “late sampling,” we found 31 metabolites that were changed at all sampling time points. The majority of these metabolites were lipids, with 20 fatty acids (mostly long-chain; three dicarboxylic; three odd-chain) and five acylcarnitines (mostly medium-chain) being significantly changed after a bout of exercise.

Thirty-eight metabolites—mostly amino acids and fatty acids—were affected in cases of early and intermediate sampling but not late sampling experiments. Ten metabolites among them mostly bile acids were affected in late and early sampling experiments. Early only changes are dominated by 19 amino acids, followed by 11 short- and medium-chain acylcarnitines, six carbohydrates, and TCA cycle intermediates, whereas intermediate changes show a variety of different metabolite groups (e.g., amino acids, nucleotides, vitamins/cofactors, and xenobiotics).

### Comparison of Metabolite Fold Changes After a Bout of Exercise

**Fig. 5:**
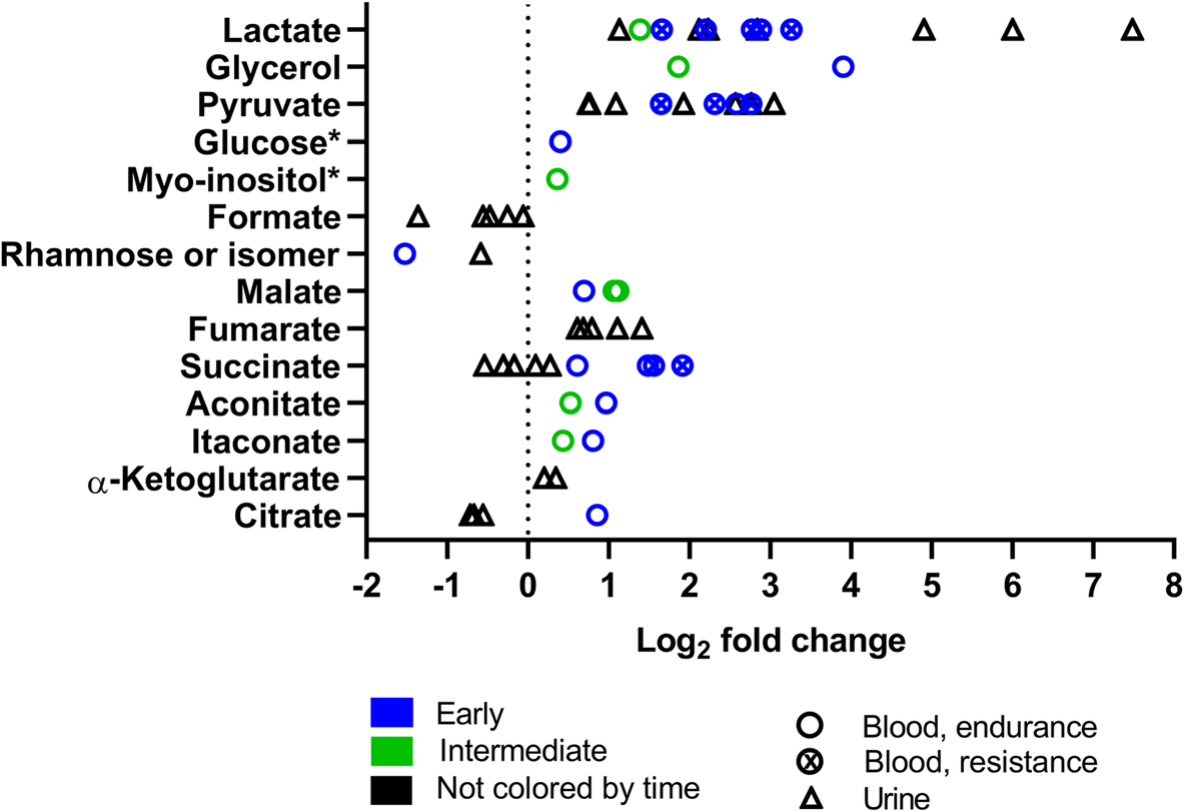
Carbohydrate and TCA cycle intermediate changes in response to exercise (log_2_ fold change versus rest). The graph shows seven metabolites of carbohydrate metabolism and seven TCA cycle intermediates reported with significant fold-changes in 20 (15 endurance, 5 resistance) and 11 (nine endurance, two resistance) experiments, respectively. One symbol represents one experiment. Rest = 0 (dotted vertical line). * fold-change values were only reported in one experiment. For detailed quantitative and qualitative changes of all carbohydrate metabolites, see Additional file 1: Table S1

**Fig. 6:**
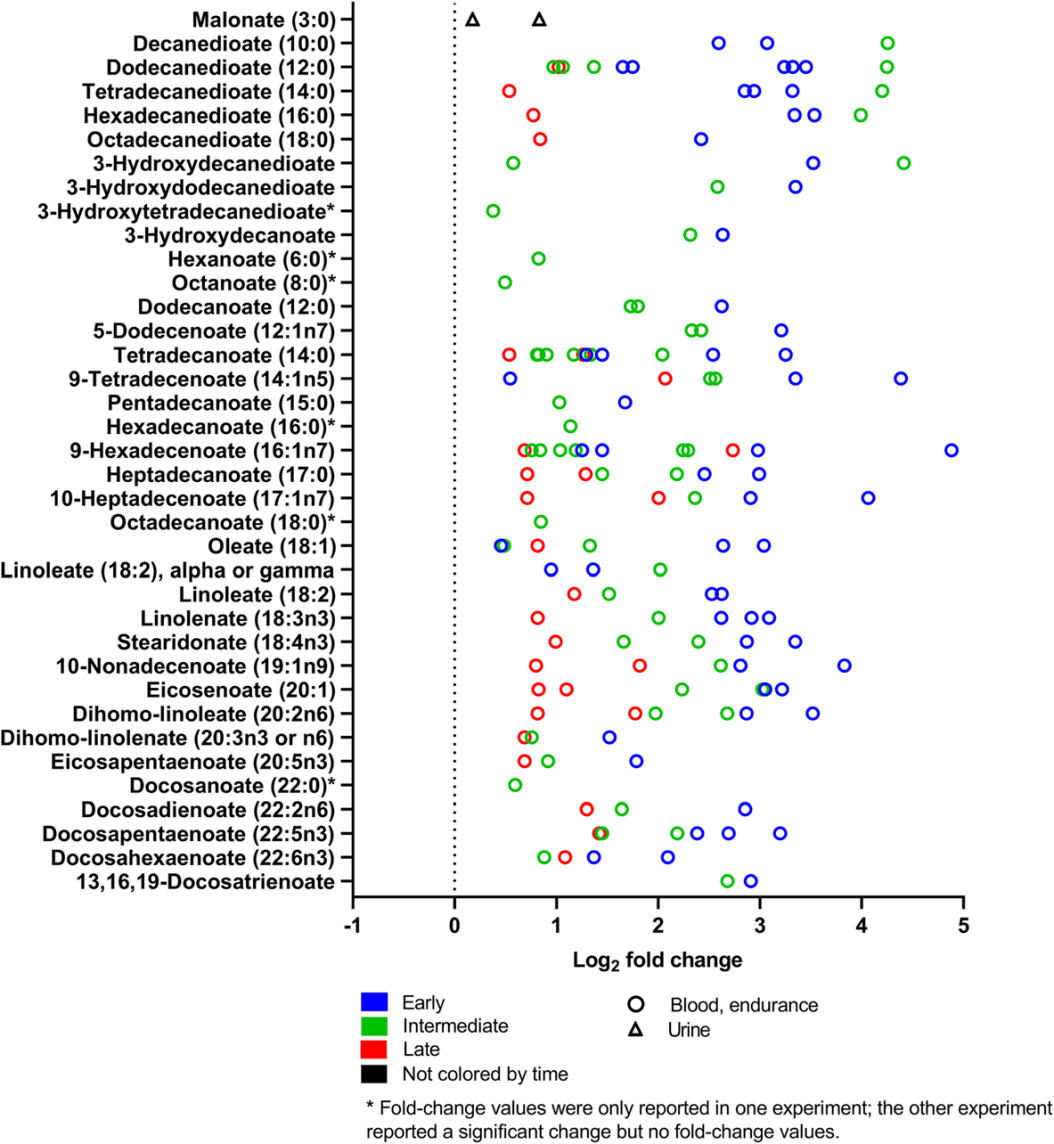
Free fatty acid changes in response to exercise (log_2_ fold change versus rest). The graph shows 37 fatty acids reported with significant fold-changes after exercise in 16 experiments (all endurance). One symbol represents one experiment. Rest = 0 (dotted vertical line). * fold-change values were only reported in one experiment. For detailed quantitative and qualitative changes of all fatty acids, see Additional file 1: Table S2

**Fig. 7:**
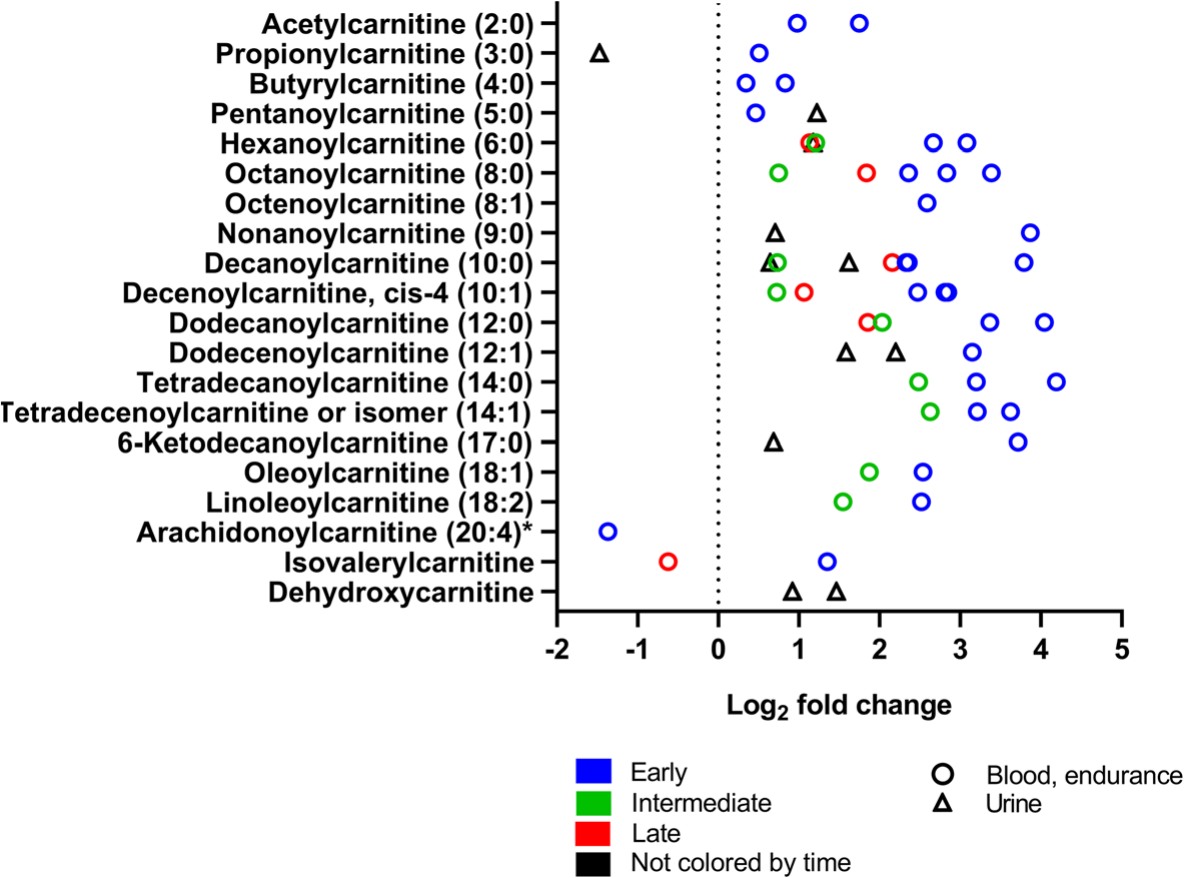
Acylcarnitine changes in response to exercise (log_2_ fold change versus rest). The graph shows twenty acylcarnitines reported with fold-changes after exercise in 10 experiments (all endurance). One symbol represents one experiment. Rest = 0 (dotted vertical line). * fold-change values were only reported in one experiment. For detailed quantitative and qualitative changes of all acylcarnitines, see Additional file 1: Table S2

**Fig. 8:**
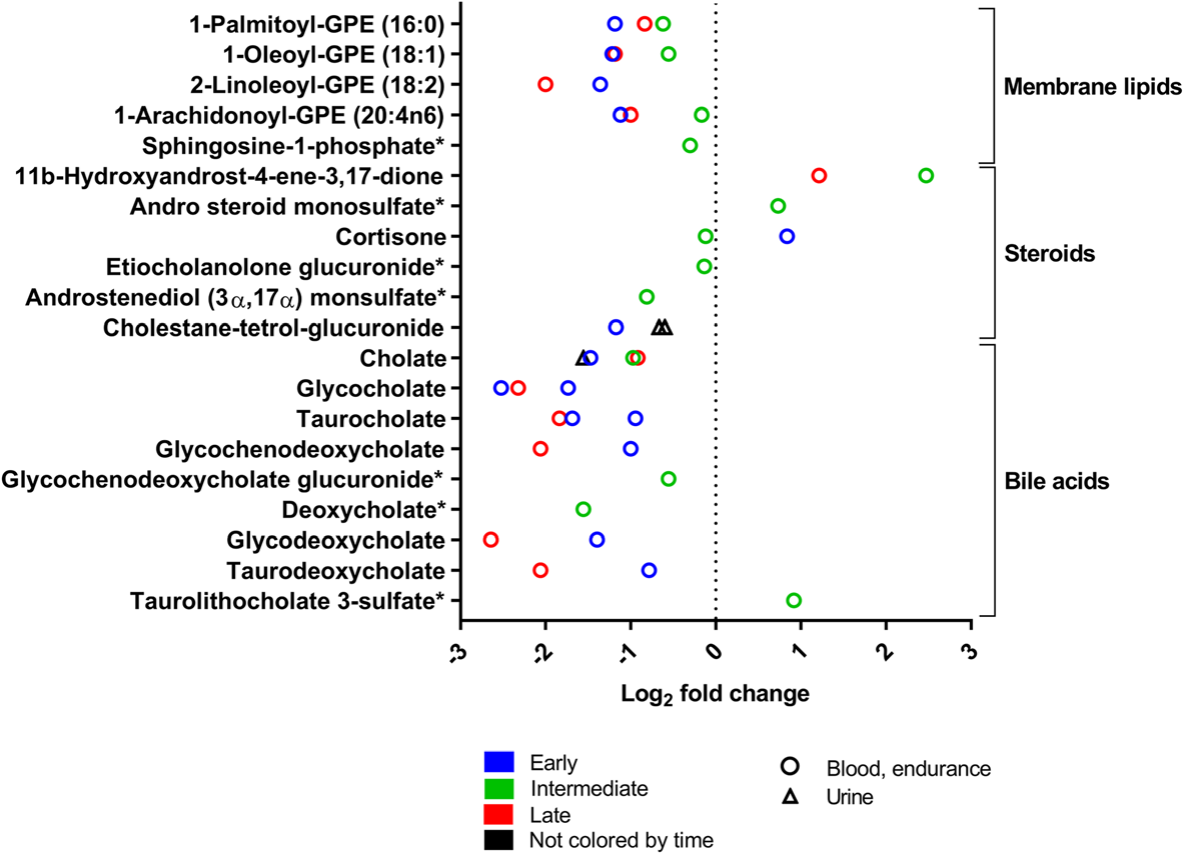
Membrane lipids, steroids and bile acid concentration changes in response to exercise (log_2_ fold change versus rest). The graph shows five membrane lipids (changed in three experiments), six steroids (changed in six experiments), and nine primary bile acids (changed in five experiments) significantly changed after exercise. Rest = 0 (dotted vertical line). One symbol represents one experiment. * fold-change values were only reported in one experiment; the other experiment(s) reported a significant change but no fold-change values. For detailed quantitative and qualitative changes of all ketone bodies, see Additional file 1: Table S2

**Fig. 9:**
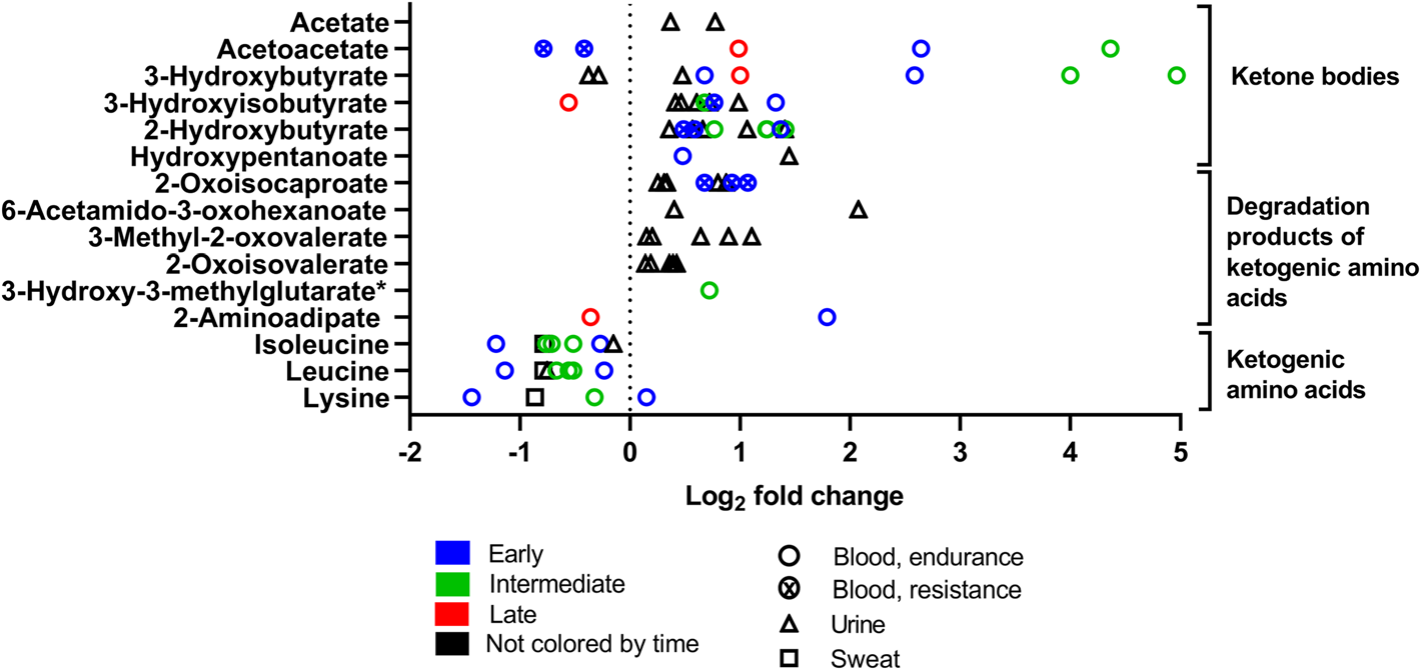
Ketone bodies, ketogenic amino acids, and their degradation products changes in response to exercise (log_2_ fold change versus rest). The graph shows 12 ketone bodies and ketogenic precursors of amino acid degradation and three ketogenic amino acids that changed significantly in 31 experiments (26 endurance, five resistance). Ketogenic amino acids are displayed for the overview but are also as part of Fig. 10. Rest = 0 (dotted vertical line). One symbol represents one experiment. * fold-change values were only reported in one experiment; the other experiment(s) reported a significant change but no fold-change values. For detailed quantitative and qualitative changes of all ketone bodies, see Additional file 1: Table S2

**Fig. 10:**
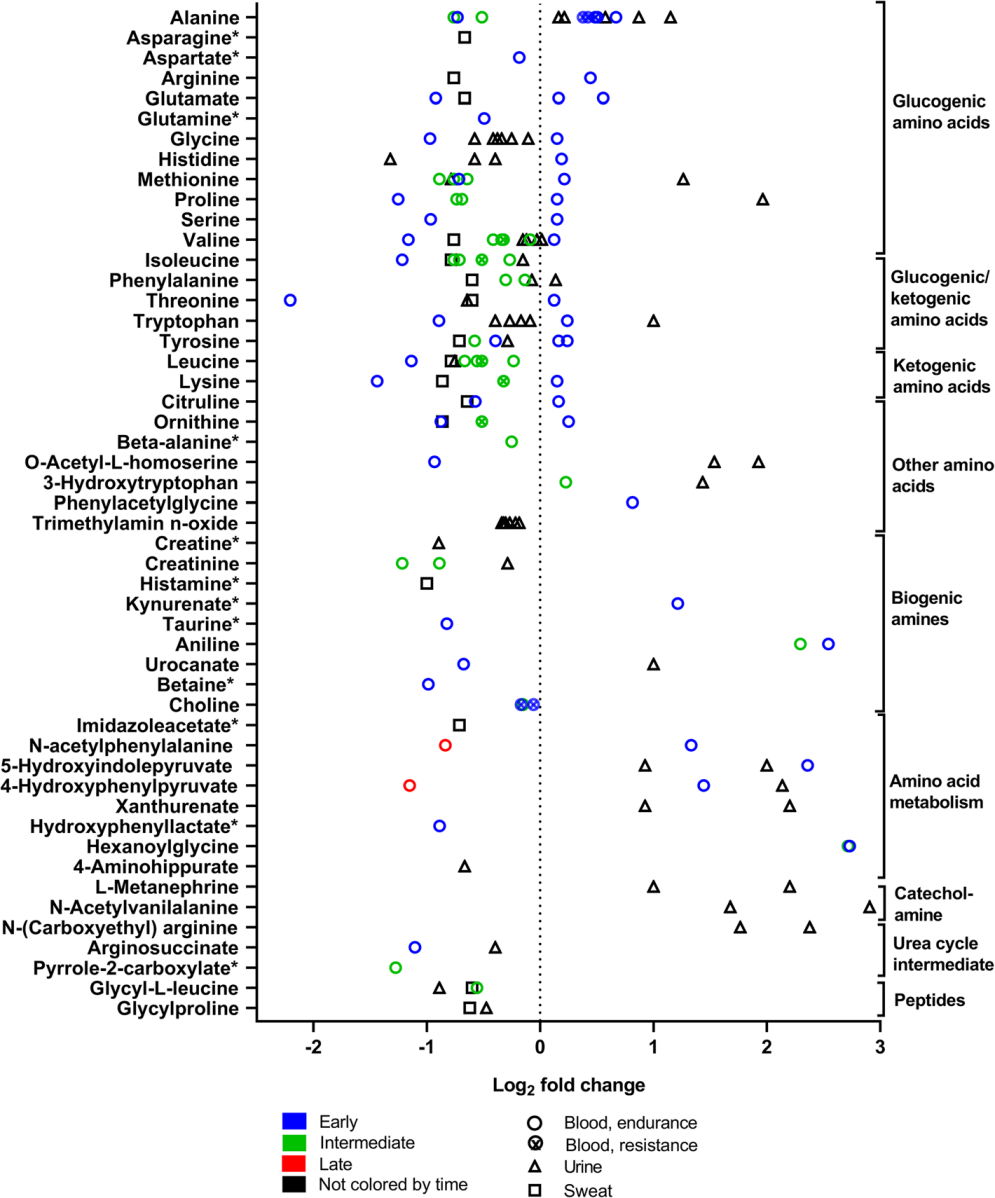
Amino acids, their derivates and peptides changes in response to exercise. The graph shows 48 amino acids, their derivates, and two peptides that significantly changed in 32 (26 endurance, six resistance) and six (all endurance) experiments, respectively, after exercise. Rest = 0 (dotted vertical line). One symbol represents one experiment. * fold-change values were only reported in one experiment; the other experiment(s) reported a significant change but no fold-change values. For detailed quantitative and qualitative changes of all amino acids, see Additional file 1: Table S3

**Fig. 11:**
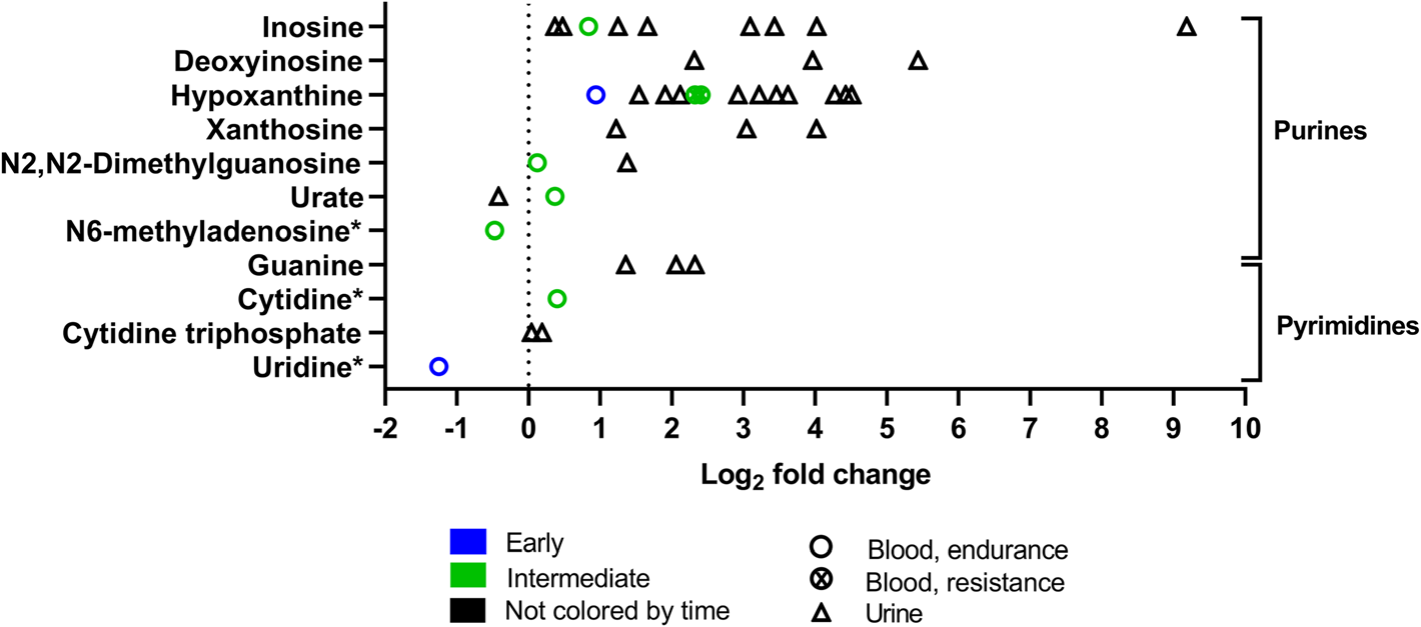
Nucleotide changes in response to exercise. The graph shows eleven nucleotides that changed significantly in 14 experiments (12 endurance, two resistance). Rest = 0 (dotted vertical line). One symbol represents one experiment. * fold-change values were only reported in one experiment; the other experiment(s) reported a significant change but no fold-change values. For detailed quantitative and qualitative changes of all nucleotides, see Additional file 1: Table S4

**Fig. 12:**
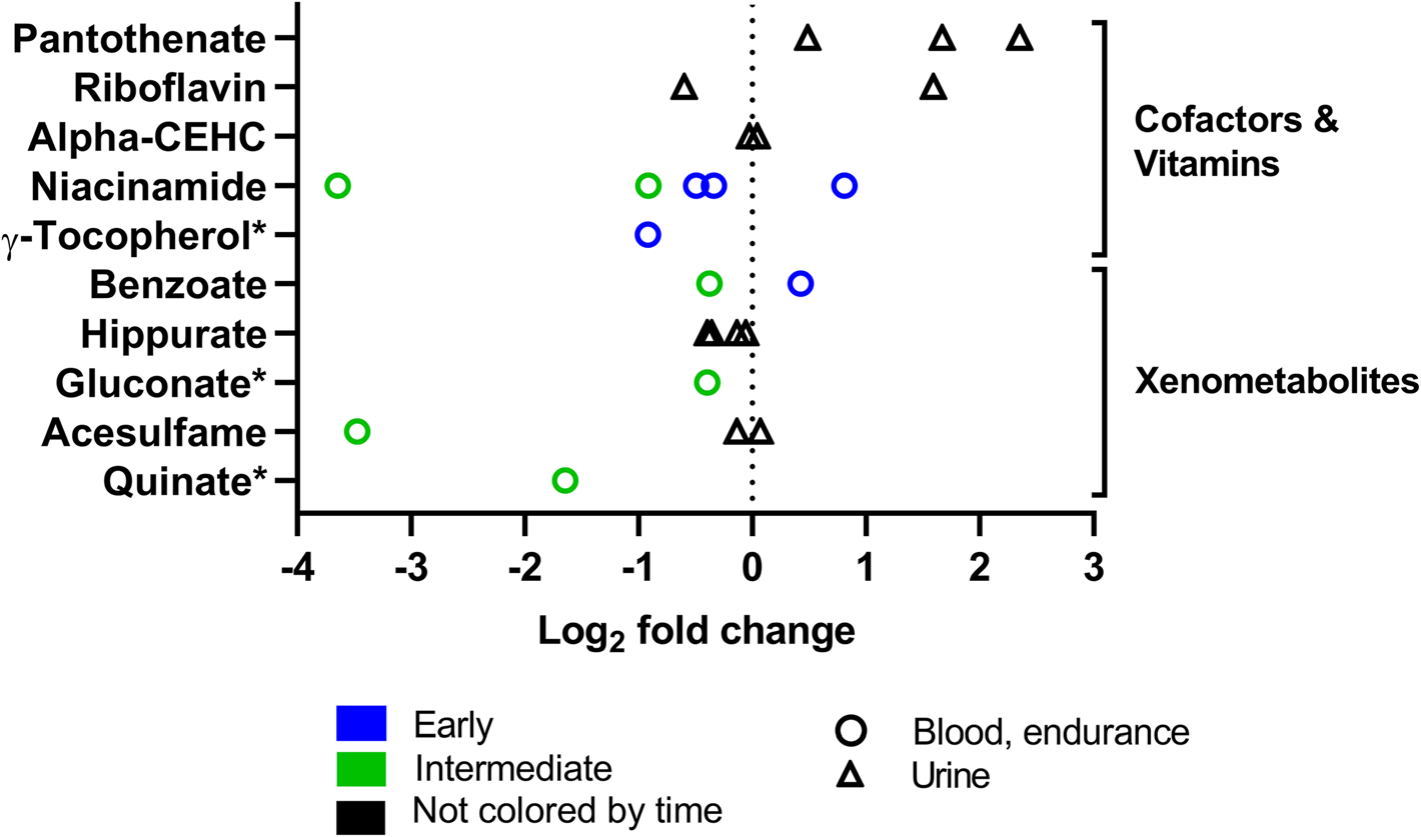
Cofactor/vitamin and xenometabolite changes in response to exercise (log_2_ fold change versus rest). The graph shows five cofactors or vitamins and six xenometabolites that changed significantly in 17 experiments (all endurance**)** Rest = 0 (dotted vertical line). One symbol represents one experiment. * fold-change values were only reported in one experiment; the other experiment reported a significant change but no fold-change values. For detailed quantitative and qualitative changes of all cofactors/vitamins and xenometabolites, see Additional file 1: Table S5

To analyze the quantitative range of metabolite effects, we summarized the fold changes of all 31 experiments reporting this information for each metabolite class (Figs. 5, 6, 7, 8, 9, 10, 11, and 12). For simplification, we pooled the results from serum, plasma, capillary blood, and non-specified blood in our overview. The rationale for this is that metabolite concentrations in human serum and plasma correlate (*r* = 0.81), with concentrations being generally higher in serum [50].

#### Carbohydrate Metabolism and TCA Cycle

Figure 5 shows carbohydrates and TCA cycle intermediates. Carbohydrates are metabolized to synthesize ATP via glycolytic lactate formation or via their oxidative phosphorylation. In the context of exercise, lactate is the most measured metabolite as its concentration at a given exercise intensity is a measure for endurance capacity [51]. The concentrations of lactate and pyruvate increase in various body fluids, as expected, whereas formate, a by-product of ketone body synthesis, and the sugar rhamnose (hexose) decrease.

In several reactions, the TCA cycle uses acetyl-CoA derived from carbohydrates, fats, or amino acids especially for nicotinamide adenine nucleotide (NADH) and subsequent ATP synthesis. After a bout of exercise, TCA cycle intermediates mainly increase in blood and urine. In blood, TCA intermediates are upregulated, especially in the early phase (until 30 min after exercise) by both endurance and resistance exercise.

#### Lipids and Lipid-Derived Compounds

Lipids are hypdrophobic molecules including fuels for energy metabolism such as triacylglycerols, signaling molecules such as steroids or phosphatidic acid, and structural components of cell membranes including phospholipids and sphingolipids. Here, we summarize the exercise-induced concentration changes of different subgroups of lipid metabolism or their derived compounds after exercise: free fatty acids, acyl-carnitines, ketone bodies, bile acids, steroids, sterols, sphingolipids, and glycerophospholipids.

Figure 6 shows the concentration changes of fatty acids. Fatty acids are carboxylic acids with an aliphatic chain and can be categorized according to their length and structure into short-, medium-, and long-chain, saturated and unsaturated fatty acids. Next to glucose, fatty acids are the major muscular energy fuel during exercise [52]. After a bout of exercise, the concentrations of various free fatty acids increase in human blood as a consequence of exercise-induced lipolysis. The majority of free fatty acid concentrations are changed most early after exercise. Contrarily, four of six dicarboxylates were reported with highest fold-changes between > 0.5 and 3 h (intermediate) after exercise.

Figure 7 shows the concentration changes of acylcarnitines. Acylcarnitines are fatty acids bound to carnitine. They are fatty acid intermediates that are transported into the mitochondria but can leave cells to appear in blood and other biofluids. Similarly to other lipids, the concentrations of almost all acylcarnitines increase in blood and urine in response to a bout of exercise. Like fatty acids, they increase especially early after exercise. In contrast to fatty acids, some acylcarnitines are also detected in urine.

Figure 8 shows exercise-induced changes in bile acids, glycerophospholipids, sphingolipids, and steroids. Bile acids are synthesized in the hepatic cytosol out of cholesterol and help to digest dietary fat in the intestine. After a bout of exercise, the concentrations of several bile acids decrease mainly in blood. The highest fold-decreases are reported in late (> 3–24 h after exercise) sampling time points. Glycerophospholipids such as glycerophosphatidylethanolamines and sphingolipids are mainly associated components of human biological membranes. Overall, these lipid classes decrease their concentration in blood and urine after a bout of exercise, decreasing highest early after exercise. Steroids especially act as steroid hormones such as testosterone or cortisol and are derived from cholesterol [53]. A bout of exercise changes several steroids in blood but there is no uniform change of concentration.

Figure 9 shows the concentration changes in ketone bodies. Ketone bodies are “energy metabolites” synthesized from acetyl-CoA or ketogenic amino acids such as leucine in the liver. Ketone bodies are used in particular in brain and muscle when carbohydrates are limited, e.g., during fasting or prolonged exercise [54].

After an acute bout of exercise, the concentration of most ketone bodies and their precursors increases significantly in different human body fluids. 3-Hydroxybutyrate and acetoacetate, the classic ketone bodies, show higher increases in intermediate samples compared to early and late samples. In resistance exercises, acetoacetate even decreased early following exercise. Other ketogenic compounds that result from the degradation of branched chain amino acid (BCAA) like 2-oxoisovalerate or 3-methyl-2-oxovalerate do not show this timing-pattern

#### Amino Acids, Peptides, and Related Metabolites

Figure 10 shows amino acids and peptide changes after exercise. Amino acids comprise 20 proteinogenic amino acids encoded by deoxyribonucleic acid (DNA), non-proteinogenic amino acids, derivates, and amino acids that are modified in proteins and then degraded into modified amino acids such as 3-methylhistidine.

Amino acids also are part of the glucose-alanine cycle. The glucose-alanine cycle degrades amino acids to supply glucose to muscles. Here, the remaining amino groups are transported to the liver in the form of alanine to generate ammonia in the urea cycle [55]. The main finding is that an acute bout of exercise changes the concentration of amino acids and their degradation products significantly in different human body fluids (Fig. 10). In contrast to the results for most lipids, the findings for many amino acids are not consistent across experiments. While similar fold changes have been observed in the same tissue for amino acids such as glycine or trimethylamine-n-oxide, vastly different changes within the same body fluid were reported for amino acids such as alanine, valine, or tryptophan.

Organic bounds between amino acid monomers form peptides, which are reported as dipeptides (compounds of two amino acids) here. They can be a part of enzymes or signaling molecules in metabolism. Within peptides, especially the dipeptides of glycine with leucine or proline decrease in serum and plasma after a bout of exercise.

#### Nucleotides

Figure 11 shows nucleotide changes after exercise. Nucleotides are organic molecules that are the substrates for both DNA and ribonucleic acid (RNA) synthesis. Moreover, nucleotides such as ATP are key metabolites for energy metabolism and nucleotides such as cyclic adenosine monophosphate (cAMP) or guanosine triphosphate (GTP) are involved in cellular signal transduction. However, phosphorylated metabolites are rarely detected in blood and other biofluids because phosphorylation traps metabolites inside cells [56]. Furthermore, nucleotides such as coenzyme A and NAD can act as mediators of hormone and cofactor reactions. After one bout of exercise, many nucleotides as well as degradation products of nucleotide catabolism such as inosine and hypoxanthine mostly increase their concentrations in human urine and blood in the early and intermediate phase following exercise.

#### Cofactors and Vitamins and Xenometabolites

Figure 12 shows changes in cofactors, vitamins, and xenometabolites after exercise. The metabolism of cofactors and vitamins contains a variety of biochemical transformations. Organic compounds of non-proteinogenic origin, including some vitamins, assist these transformations. Observed changes of cofactors and vitamins differ between the here summarized experiments. Like cofactors and vitamins, xenometabolites are exogenous compounds. Xenometabolites can be drugs, food ingredients such as preservatives, plant components, or pesticides. Xenometabolites mostly decrease after a bout of exercise.

## Discussion

In this review, we summarize how metabolite concentrations change in human blood and other biofluids within 24 h after a bout of exercise. Our analysis provides the first overview of results across metabolomics studies that use different human subjects, endurance, and resistance exercise; analyze different body fluids; utilize several analysis methods; and collect samples at different time points after exercise. Even though there are many differences in-between studies, the concentrations of many metabolites such as fatty acids or acylcarnitines often change similarly after exercise. There are, however, exceptions where metabolite concentrations change in different directions after exercise. This combined dataset illustrates such differences and may help researchers to identify the causes.

### Exercise Alters the Concentrations of Metabolites that Are Involved in Energy Metabolism

Exercise is a major challenge to the body’s homeostasis as it requires an immediate, large increase of ATP re-synthesis. As a consequence, the flux of many energy metabolism reactions changes quickly with the onset of exercise. This changed flux then alters the blood and biofluid concentrations of metabolites involved in these reactions. These concentration changes reflect the mobilization, utilization, and conversion of energy metabolites such as carbohydrates and triacylglycerols (fats) to meet the ATP demand of the exercising muscles. Exercise studies also confirm that ketone bodies are generated, and amino acids are converted into glucose when carbohydrates are limited.

In the summarized studies, many of the metabolites that increased globally after exercise are lipids or related to lipid metabolism. These metabolites include glycerol (Fig. 5), free fatty acids (Fig. 6), and acylcarnitines (Fig. 7). During exercise, lipases split the triacylglycerols stored in adipose tissue into fatty acids and glycerol [57]. The fatty acids and glycerol are then released into the bloodstream, before being taken up and utilized for ATP synthesis by the exercising muscles. A new insight of this combined analysis is that all free fatty acids increase within 24 h after exercise no matter whether they are unsaturated or saturated, short, medium, or long. The earlier the post-exercise sample is taken, the higher fatty acid increases in blood are (Fig. 6) [58].

Fatty acids that are taken up by muscle are then transported into the mitochondria in several steps that involve carnitine and the formation of acylcarnitines [52]. Even though acylcarnitines are formed within the cell, increased concentrations of acylcarnitines are detected after exercise in blood and other biofluids (Fig. 7).

During high-intensity exercise, blood glucose and muscle glycogen become the dominant sources of energy [59, 60]. They enter glycolysis and as a consequence, pyruvate and lactate concentrations are increased especially during and after high intensity exercise (Fig. 5). Blood pyruvate and lactate then decline within an hour after exercise but high urine concentrations are also measured 24 h after exercise [36, 37]. In contrast to pyruvate and lactate, glycolytic intermediates did only appear in blood in one study [34] and are therefore not shown in the graphs. Normally, glycolytic intermediates, which are phosphorylated are trapped inside cells [56].

Also, at high-intensity exercise, TCA cycle flux and the concentrations of TCA cycle metabolites such as malate increase (Fig. 5) which has been previously discussed in a review [52, 61]. In our analysis, the TCA cycle intermediates succinate and malate increased most in blood after high-intensity endurance and high load resistance exercise [40, 47] especially early after exercise (Fig. 5).

When carbohydrates run out during prolonged exercise or when fasted, then especially the liver is synthesizing new substrates for energy metabolism through ketogenesis and gluconeogenesis [62]. Liver synthesizes ketone bodies from ketogenic amino acids such as leucine or lysine and glucose from glucogenic amino acids such as valine or glycine [54]. The main ketone bodies 3-hydroxybutyrate and acetoacetate are then released into the blood [54] (Fig. 9) which explains their increased blood concentration after exercise. In parallel, the degradation products such as 2-oxoisovalerate increase too (Fig. 9). For gluconeogenesis, glucogenic amino acids are degraded to pyruvate and then transaminated to alanine. Alanine goes into the blood stream and blood alanine concentration increases (Fig. 10). In the liver, it is transformed to pyruvate again, and finally into glucose. Furthermore, the degradation products of glucogenic amino acids such as n-acetylphenylalanine and 4-hydroxyphenylpyruvate increase (Fig. 10).

### After Exercise, the Concentrations of Nucleotide Degradation Products Increase, Whereas Bile Acid and Complex Lipid Concentrations Decrease

While nucleotides such as ATP or inosine monophosphate (IMP) molecules are trapped within cells [56], their unphosphorylated degradation products inosine, hypoxanthine, xanthine, and uric acid are detected in blood and other biofluids. After exercise, the concentrations of these nucleotide degradation products generally increases, especially in urine (Fig. 11). Generally, the concentrations of nucleotide degradation products increase most after high-intensity exercise [63] and most concentration changes occur between > 0.5 and 3 h after exercise [31, 43] (Figs. 4 and 11).

Exercise also lowers the concentrations of bile acids (Fig. 8). The primary bile acids cholic acid (cholate) and chenodeoxycholic acid are synthesized in the liver and secondary bile acids are then formed by intestinal bacteria [64]. The decrease of bile acids after exercise reported by metabolomics studies is in line with recent literature showing that both endurance and resistance exercise decrease the total bile acid concentration [65].

Fasting alone decreases bile acid concentrations [66] and bile acids decreased after exercise (Fig. 8), during which subjects did not eat. Therefore, lower bile acid concentrations after exercise are a result from exercise and fasting and not exercise alone.

Given that bile acid concentrations are associated with metabolic disease [67], this may identify exercise as an intervention that can modulate bile acid concentrations for therapeutic gains.

Additionally, complex lipids like glycerolipids and sphingolipids all decrease after exercise (Fig. 8). These lipids are not only important constituents of membranes but engage in signal transduction. For example, sphingosine-1-phosphate is released from cells by cell-specific transporters into the circulation. There, it can bind to five G-protein coupled receptors to regulate cellular behaviors such as survival and proliferation [68], e.g., through the modulation of Hippo signaling [69]. Whether the observed drop of sphingosine-1-phosphate after exercise (Fig. 8) can be exploited for the treatment of disease is currently unknown.

### Different Study Protocols and Feeding Can Influence Metabolite Changes

Next to many consistent metabolite changes across studies, this analysis also showed metabolite changes in different directions between studies.

One example for variable concentration changes after exercise are amino acids and their derivates. Specifically, out of 53 amino acids, 37 changed in different directions after exercise (Figs. 3 and 10). One reason for these differences could be that amino acids are used or synthesized by many reactions and that these reactions may differ between different exercise and feeding protocols. For example, amino acids can contribute up to 10% to oxidative phosphorylation [70]. Amino acids are also used as substrates for gluconeogenesis, ketogenesis [54], and protein synthesis especially after resistance exercise [71] (Fig. 10). The use of amino acids in all of these reactions will lower their concentration. Conversely, amino acids are generated by protein breakdown via the proteasome or autophagy [72] or enter the blood when proteins are digested and together breakdown and protein ingestion will increase the concentrations of amino acids in blood.

Compared to other metabolites such as fatty acids, post-exercise sample timing alone did not influence the variable changes in amino acids between the studies. What differs the most between studies is the duration of the exercise. The two studies [23, 31, 40, 47] that had the biggest difference in protocols and energy demand (a VO_2max_ test of ~ 10–15 min versus a simulated ultra-marathon of ~ 8–9 h) had the highest differences in fold-change after exercise (Fig. 10). Amino acid concentrations were lower after exercising with moderate intensity and for long duration. In contrast, amino acid concentrations were higher after exercise with high intensity but short duration.

Another factor that can influence amino acid concentrations and many other metabolites is pre- or post-exercise feeding. Especially carbohydrate intake reduces gluconeogenesis [66] and ketogenesis [54] and thereby reduces the usage of amino acids in these reactions so that the concentrations of these amino acids change less if subjects ingest carbohydrates.

## Limitations

This systematic review has limitations. First, the studies summarized in this review combine many different exercise modes with variable intensity and duration and further vary in their nutrition and sampling times. Moreover, subjects are men and women, differing in their sex hormone concentrations and in the concentration of roughly one-third of all metabolites [73]. Furthermore, subjects were of different ages, differentially trained, and may have varied in their health and body composition. This is a key source of variation in this dataset. Thus, if metabolites such as fatty acids (Fig. 6) all increase their average concentration in response to different exercise modes and in different subjects then this suggests that the increase of fatty acid concentrations is a robust response to exercise.

A second limitation of our analysis is that not all included studies measured the same set of metabolites. When we report metabolites that only change in one of the three sampling phases (early, intermediate, late), it can be that this metabolite was measured only at this specific time point after exercise.

A third limitation is that both mass spectrometry and nuclear magnetic resonance methods have been used to measure metabolites. Also, since 2010, the metabolomics protocols have become more sensitive, allowing to detect and better quantify more metabolites. Thus, variable methodology is another source of variation in this combined analysis. We have made no attempt to control for the methods used but have indicated the methods used in Additional file 2: Table S6.

A fourth limitation is that this study reports average changes, not individual changes. This is an important limitation, because individual resting blood metabolite concentrations vary greatly in-between individuals and are strongly dependent on DNA sequence variation [74]. Moreover, the response of metabolites to exercise training varies too [75] and this individual variability is not reflected in this dataset as we only report mean concentration changes.

## Conclusion and Outlook

Across different exercise modes and in different subjects, exercise often consistently changes the average concentrations of metabolites involved in energy metabolism and other branches of metabolism. This dataset should therefore be a useful resource for those that wish to study human exercise metabolism.

For the future, one important focus should be to use metabolomics to investigate whether individual metabolite concentrations or “metabolite fingerprints” (i.e., combinations of metabolites) are biomarkers for disease, metabolic function, trainability, or other “hard to measure” traits such as muscle fiber percentages. Here, it may be essential, similar to cardiovascular stress tests [76], to activate a system by exercise, as the capacity and function of many systems can only be assessed when the system is active and under stress. Many metabolic enzymes are inactive at rest and only become activated by exercise [77]. Therefore, the capacity of these enzymes may only be revealed by measuring metabolite concentrations during and after an exercise challenge. The best known example for this paradigm is of course lactate, as resting concentrations do not but exercise lactate concentrations do predict the capacity of aerobic metabolism [51].

Finally, while metabolite concentrations might be useful indicators of health or fitness-related phenotypes, they often do not report the flux or capacity of metabolic reactions. Here, the combination of stable isotope-labeled tracer molecules such as glucose or amino acids in combination with mass spectrometry analysis may in future allow the measurement of metabolic flux and this has been termed fluxomics [78]. Applying this technology to exercise studies is arguably the next methodological frontier of metabolic research in relation to sport and exercise.

## Additional Files

**Additional file 1: Table S1.** Carbohydrate Metabolites and TCA cycle intermediates. **Table S2.** Lipids and intermediates of lipid metabolism. **Table S3.** Amino Acids and Peptides. **Table S4.** Nucleotides. **Table S5**. Cofactors/Vitamins and Xenometabolites.

**Additional file 2: Table S6.** Descriptive summary of 57 experiments that reported metabolites concentration changes after a bout of exercise.

**Additional file 3: Table S7.** Metabolites changed in relation to time point of sampling (with Fig. 4).

## Data Availability

All data generated or analyzed during this study are included in this published article [and its supplementary information files].

## Abbreviations

ATP: Adenosine triphosphate
BCAA: Branched chain amino acid
BMI: Body mass index, calculated with bodyweight divided by body height (kg/m^2^)
cAMP: Cyclic adenosine monophosphate
DNA: Deoxyribonucleic acid
GTP: Guanosine triphosphate
H-NMR: Proton nuclear magnetic resonance
IMP: Inosine monophosphate
kDA: Kilo Dalton
mM: Millimolar
NADH/NAD: Nicotinamide adenine nucleotide
PICO: Population Intervention Control Outcome
RNA: Ribonucleic acid
TCA cycle: Tricarboxylic acid cycle
VO_2max_: Maximum oxygen uptake capacity
