# Supplementary material for: Metabolite Concentration Changes in Humans After a Bout of Exercise: a Systematic Review of Exercise Metabolomics Studies
Source: Sports Med Open. 2020 Feb 10;6:11. doi: 10.1186/s40798-020-0238-4 (PMC7010904; doi:10.1186/s40798-020-0238-4)
Supplement: Supplementary file 1 — Additional file 1: Table S1. Carbohydrate Metabolites and TCA cycle intermediates. Table S2. Lipids and intermediates of lipid metabolism. Table S3. Amino Acids and Peptides. Table S4. Nucleotides. Table S5. Cofactors/Vitamins and Xenometabolites. [file 40798_2020_238_MOESM1_ESM.docx]

**Supplementary Data, Tables S1-5**

Significant fold changes and decreases/increases of metabolites after a bout of exercise in 57 experiments. This table combines the results from the graphs (31 studies with fold-changes reported, **c)**) and from 26 experiments that only reported if the metabolite decreased or increased after exercise. The summary is described in columns **d)** and **e)**.

**Table S1.** **Carbohydrate Metabolites and TCA cycle intermediates**

| **a)**  Pathway | **b)**  Metabolite | **c)**  In 31 experiments n changes were reported. | | | **d)**  In all 57 experiments n increases were reported | | **e)**  In all 57 experiments n decreases were reported | |
| --- | --- | --- | --- | --- | --- | --- | --- | --- |
|  |  | MFC | SD | n | n | References | n | References |
| **Carbohydrate Metabolism** | Lactate | 25.01 | 49.35 | 13 | 28 ↑ | Pechlivanis, 2015 (2); Hall, 2016; Messier, 2017; Mukherjee, 2014 (2); Pechlivanis, 2010 (2); Berton, 2016 (3); Enea, 2010; Lewis 2010 (5); Coelho, 2016 (2); Muhsen, 2016; Danaher, 2015 (3); Valerio, 2017 (2); Zafeiridis, 2016; Sun, 2017; Peake, 2014 | - |  |
|  | Glycerol | 9.32 | 8.03 | 2 | 8 ↑ | Nieman, 2015 (2); Messier, 2017 (2); Lewis, 2010 (3); Zafeiridis, 2016 | - |  |
|  | Pyruvate | 4.66 | 2.30 | 11 | 19 ↑ | Daskalaki, 2015 (2); Pechlivanis, 2015; Mukherjee, 2014 (2); Pechlivanis, 2010 (2); Berton, 2016 (2); Enea, 2010; Lewis, 2010 (5); Valerio, 2017 (2); Zafeiridis, 2016; Sun, 2017 | - |  |
|  | Glucose-6-phosphate | - | - | - | 4 ↑ | Ra, 2014; Lewis, 2010 (3) | - |  |
|  | Glucose | 1.32 | - | 1 | 3 ↑ | Breit, 2015; Zauber, 2012; Zafeiridis, 2016 | 1 ↓ | Messier, 2017 |
|  | Myo-inositol | 1.29 | 0.00 | 1 | 2 ↑ | Karl, 2017; Zauber, 2012 | - |  |
|  | Erythronate | - | - | - | - |  | 2 ↓ | Danaher, 2015 (2) |
|  | Formate | 0.72 | 0.21 | 5 | - |  | 6 ↓ | Pechlivanis, 2015; Mukherjee, 2014 (2); Pechlivanis, 2010 (2); Sun, 2017 |
|  | Fructose | - | - | - | 2 ↑ | Zauber, 2012 (2) | - |  |
|  | Mannitol | - | - | - | 2 ↑ | Zauber, 2012 (2) | - |  |
|  | Isomaltose | - | - | - | 2 ↑ | Zauber, 2012 (2) | - |  |
|  | Beta-D-Methylglucopyranoside | - | - | - | 2 ↑ | Chorell, 2011 (2) | - |  |
|  | Rhamnose or isomer | 0.51 | 0.23 | 2 | - |  | 3 ↓ | Muhsen, 2016; Howe, 2018; Prado, 2017 |
| **TCA cycle** | Malate | 1.96 | 0.30 | 3 | 13 ↑ | Peake, 2014 (3); Zauber, 2012; Chorell, 2011; Lewis, 2010 (5); Danaher, 2015 (3) | - |  |
|  | Fumarate | 1.94 | 0.47 | 5 | 11 ↑ | Pechlivanis, 2015; Mukherjee, 2014 (2); Pechlivanis, 2010 (2); Lewis, 2010 (5); Sun, 2018 | - |  |
|  | Succinate | 1.75 | 1.13 | 9 | 16 ↑ | Pechlivanis, 2010 (2); Berton, 2016; Valerio, 2017; Peake, 2014 (2); Hall, 2016; Messier, 2017 (2); Enea, 2010; Lewis, 2010 (5); Zafeiridis, 2016 | 3 ↓ | Pechlivanis, 2015; Mukherjee, 2014 (2) |
|  | Aconitate | 1.7 | 0.08 | 2 | 5 ↑ | Peake, 2014 (2); Lewis, 2010 (3) | - |  |
|  | Itaconate | 1.55 | 0.28 | 2 | 2 ↑ | Peake, 2014 (2) | - |  |
|  | Alpha-Ketoglutarate | 1.21 | 0.08 | 2 | 4 ↑ | Pechlivanis, 2010 (2); Zafeiridis, 2016; Lewis, 2010 | - |  |
|  | Citrate | 0.93 | 0.59 | 4 | 10 ↑ | Peake, 2014 (HIT); Zauber, 2012 (2); Chorell, 2011; Lewis, 2010 (3); Danaher, 2015 (2); Zafeiridis, 2016 | 4 ↓ | Pechlivanis, 2015; Pechlivanis, 2010 (2); Sun, 2018 |

**Table S2. Lipids and lipid metabolism.**

| **a)**  Pathway | **b)**  Metabolite | **c)**  In 31 experiments n changes were reported. | | | **d)**  In all 57 experiments n increases were reported | | **e)**  In all 57 experiments n decreases were reported | |
| --- | --- | --- | --- | --- | --- | --- | --- | --- |
|  |  | MFC | SD | n | n | References | n | References |
| **Fatty acid, Dicarboxylate** | Malonate (3:0) | 1.46 | 0.46 | 2 | 2 ↑ | Mukherjee, 2014 (2) | - |  |
|  | Decanedioate (10:0) | 11.18 | 6.96 | 3 | 3 ↑ | Howe, 2018; Nieman, 2015 (2) | 1 ↓ | Prado, 2017 |
|  | Dodecanedioate (12:0) | 10.28 | 6.03 | 5 | 5 ↑ | Howe, 2018; Nieman, 2015 (2); Nieman, 2013 (2) | - |  |
|  | Tetra-Decanedioate (14:0) | 8.95 | 6.15 | 5 | 5 ↑ | Howe, 2018; Nieman, 2015 (2); Nieman, 2013 (2) | 1 ↓ | Prado, 2017 |
|  | Hexadecanedioate (16:0) | 9.84 | 5.94 | 4 | 4 ↑ | Nieman, 2015 (2); Nieman, 2013 (2) | 1 ↓ | Prado, 2017 |
|  | Octadecanedioate (18:0) | 3.58 | 2.52 | 2 | 2 ↑ | Nieman, 2013 (2) | - |  |
| **Fatty acid, Monohydroxy** | 3-Hydroxydecanedioate | 11.43 | 9.91 | 3 | 3 ↑ | Karl, 2017; Nieman, 2015 (2) | 1 ↓ | Prado, 2017 |
|  | 3-Hydroxydodecanedioate | 8.10 | 2.98 | 2 | 2 ↑ | Nieman, 2015 (2) | - |  |
|  | 3-Hydroxytetradecanedioic acid | 1.30 | - | 1 | 1 ↑ | Karl, 2017 | 1 ↓ | Prado, 2017 |
|  | 3-Hydroxydecanoate | 5.60 | 0.87 | 2 | 2 ↑ | Nieman, 2015 (2) | 1 ↓ | Prado, 2017 |
|  | CH2 fatty acids (ẟ 1.29; ẟ 1.57) | - | - | - | - |  | 2 ↓ | Messier, 2017 (2) |
|  | Fatty acids (ẟ 2.01) | - | - | - | 1 ↑ | Hall, 2016 | 2 ↓ | Messier, 2017 (2) |
| **Short chain fatty acids** | Butyrate (4:0) | - | - | - | 1 ↑ | Prado, 2017 | 1 ↓ | Chorell, 2011 |
| **Medium chain fatty acids** | Hexanoate (6:0) | 1.41 | - | 1 | 1 ↑ | Karl, 2017 | - |  |
|  | Octanoate (8:0) | 1.77 | - | 1 | 1 ↑ | Karl, 2017 | 1 ↓ | Prado, 2017 |
|  | Dodecanoate (12:0) | 3.26 | 1.31 | 8 | 8 ↑ | Karl, 2017; Nieman, 2015 (2); Peake, 2014 (5) | 2 ↓ | Danaher, 2015 (2) |
| **Long chain,**  **very long chain and polyunsaturated fatty acids** | Tetradecanoate (14:0) | 3.22 | 2.32 | 12 | 12 ↑ | Karl, 2017; Nieman, 2015 (3); Nieman, 2013 (2); Peake, 2014 (6) | - |  |
|  | Pentadecanoate (15:0) | 2.62 | 0.81 | 2 | 2 ↑ | Karl, 2017; Howe, 2018 | 1 ↓ | Prado, 2017 |
|  | Hexadecanoate (16:0) | 2.20 | - | 1 | 2 ↑ | Karl, 2017; Prado, 2017 | 2 ↓ | Danaher, 2015 (2) |
|  | Heptadecanoate (17:0) | 4.13 | 2.35 | 6 | 6 ↑ | Karl, 2017; Nieman, 2015 (3); Nieman, 2013 (2) | - |  |
|  | Octadecanoate (18:0) | 1.80 | - | 1 | 1 ↑ | Karl, 2017 | 2 ↓ | Danaher, 2015 (2) |
|  | Docosanoate (22:0) | 1.51 | - | 1 | 2 ↑ | Karl, 2017; Prado, 2017 | - |  |
|  | Oleate (18:1) | 3.58 | 2.92 | 6 | 6 ↑ | Karl, 2017; Howe, 2018; Nieman, 2013 (2); Peake, 2014 (2) | - |  |
|  | Eicosenoate (20:1) | 5.73 | 3.31 | 6 | 6 ↑ | Karl, 2017; Nieman, 2015 (3); Nieman, 2013 (2) | 1 ↓ | Prado, 2017 |
|  | Linoleate (alpha or gamma) | 2.85 | 1.09 | 3 | 3 ↑ | Karl, 2017; Peake, 2014 (2) | - |  |
|  | Linoleate (18:2) | 4.26 | 1.99 | 4 | 4 ↑ | Karl, 2017; Howe, 2018; Nieman, 2013 (2) | 1 ↓ | Chorell, 2011 |
|  | Linolenate (18:3n3) | 5.6 | 2.73 | 5 | 5 ↑ | Howe, 2018; Nieman, 2015 (2); Nieman, 2013 (2) | - |  |
|  | Stearidonate (18:4n3) | 5.58 | 3.28 | 5 | 5 ↑ | Karl, 2017; Nieman, 2015 (2); Nieman, 2013 (2) | - |  |
|  | Dihomo-linolenate (20:3n3 or n6) | 2.06 | 0.71 | 3 | 3 ↑ | Karl, 2017; Nieman, 2013 (2) | - |  |
|  | Eicosapentaenoate (EPA; 20:5n3) | 2.32 | 0.99 | 3 | 3 ↑ | Karl, 2017; Nieman, 2013 (2) | - |  |
|  | Docosapentaenoate (n3 DPA; 22:5n3) | 5.14 | 2.46 | 6 | 6 ↑ | Karl, 2017; Howe, 2018; Nieman, 2015 (2); Nieman, 2013 (2) | 1 ↓ | Prado, 2017 |
|  | Docosahexaenoate (DHA; 22:6n3) | 2.7 | 1.09 | 4 | 4 ↑ | Karl, 2017; Howe, 2018; Nieman, 2013 (2) | - |  |
|  | 9-Tetradecaenoate (14:1n5) | 8.06 | 6.90 | 6 | 6 ↑ | Karl, 2017; Nieman, 2015 (2); Nieman, 2013 (2); Peake, 2014 | - |  |
|  | Dihomo-linoleate (20:2n6) | 5.71 | 3.47 | 6 | 6 ↑ | Karl, 2017; Nieman, 2015 (3); Nieman, 2013 (2) | - |  |
|  | Docosadienoate (22:2n6) | 4.27 | 2.58 | 3 | 3 ↑ | Karl, 2017; Nieman, 2013 (2) | - |  |
|  | 5-Dodecenoate (12:1n7) | 6.55 | 2.35 | 3 | 3 ↑ | Karl, 2017; Nieman, 2015 (2) | 1 ↓ | Prado, 2017 |
|  | 13,16,19-Docosatrienoate | 6.97 | 0.78 | 2 | 2 ↑ | Nieman, 2015 (2) | - |  |
|  | 9-Hexadecenoate (16:1n7) | 8.23 | 11.81 | 13 | 13 ↑ | Karl, 2017; Howe, 2018; Nieman, 2015 (3); Nieman, 2013 (2); Peake, 2014 (6) | 1 ↓ | Prado, 2017 |
|  | 10-Heptadecenoate (17:1n7) | 7.01 | 5.84 | 5 | 5 ↑ | Nieman, 2015 (3); Nieman, 2013 (2) | - |  |
|  | 10-Nonadecenoate (19:1n9) | 6.53 | 4.79 | 5 | 5 ↑ | Nieman, 2015 (3); Nieman, 2013 (2) | - |  |
|  | CH3 fatty acids (d 0.9) | - | - | - | - |  | 2 ↓ | Messier, 2017 (2) |
|  | Double bond fatty acids (ẟ 5.32) | - | - | - | - |  | 2 ↓ | Messier, 2017 (2) |

**Table S2 continued**

| **a)**  Pathway | **b)**  Metabolite | **c)**  In 31 experiments n changes were reported. | | | **d)**  In all 57 experiments n increases were reported | | **e)**  In all 57 experiments n decreases were reported | |
| --- | --- | --- | --- | --- | --- | --- | --- | --- |
|  |  | MFC | SD | n | n | Reference | n | Reference |
| **Acylcarnitines** | Acetylcarnitine (2:0) | 2.66 | 0.98 | 2 | 4 ↑ | Breit, 2015; Howe, 2018; Prado, 2017; Hall, 2016 | - |  |
|  | Propionylcarnitine (3:0) | 0.89 | 0.75 | 2 | 1 ↑ | Howe, 2018 | 1 ↓ | Muhsen, 2016 |
|  | Butyrylcarnitine (4:0) | 1.52 | 0.36 | 2 | 2 ↑ | Breit, 2015; Howe, 2018 | 1 ↓ | Prado, 2017 |
|  | Pentanoylcarnitine (5:0) | 1.86 | 0.67 | 2 | 2 ↑ | Daskalaki, 2015; Breit, 2015 | - |  |
|  | Hexanoylcarnitine (6:0) | 4.31 | 2.91 | 5 | 7 ↑ | Daskalaki, 2015; Nieman, 2015 (2); Nieman, 2013 (2); Lehmann, 2010 | - |  |
|  | Octanoylcarnitine (8:0) | 5.59 | 3.37 | 5 | 7 ↑ | Howe, 2018; Nieman, 2015 (2); Nieman, 2013 (2); Lehmann, 2010 (2) | - |  |
|  | Octenoylcarnitine (8:1) | 6.01 | - | 1 | 2 ↑ | Lehmann, 2010; Howe, 2018 | - |  |
|  | Nonanoylcarnitine (9:0) | 8.11 | 9.16 | 2 | 2 ↑ | Muhsen, 2016; Howe, 2018 | - |  |
|  | Decanoylcarnitine (10:0) | 4.96 | 4.18 | 7 | 9 ↑ | Daskalaki, 2015; Muhsen, 2016; Howe, 2018; Nieman, 2015 (2); Nieman, 2013 (2); Lehmann, 2010 (2) | 1 ↓ | Prado, 2017 |
|  | Decenoylcarnitine, cis-4 (10:1) | 4.70 | 2.66 | 5 | 5 ↑ | Howe, 2018; Nieman, 2015 (2); Nieman, 2013 (2) | - |  |
|  | Dodecanoylcarnitine (12:0) | 8.62 | 6.06 | 4 | 4 ↑ | Nieman, 2015 (2); Nieman, 2013 (2) | - |  |
|  | Dodecenoylcarnitine (12:1) | 5.48 | 3.02 | 3 | 5 ↑ | Daskalaki, 2015 (2); Howe, 2018; Lehmann, 2010 (2) | 1 ↓ | Prado, 2017 |
|  | Tetradecanoylcarnitine (14:0) | 12.25 | 4.93 | 3 | 4 ↑ | Lehmann, 2010; Howe, 2018; Nieman, 2015 (2) | - |  |
|  | Tetradecenoylcarnitine/isomer (14:1) | 8.00 | 1.71 | 3 | 4 ↑ | Lehmann, 2010; Howe, 2018; Nieman, 2015 (2) | - |  |
|  | 6-Ketodecanoylcarnitine (17:0) | 7.37 | 8.14 | 2 | 2 ↑ | Muhsen, 2016; Howe, 2018 | - |  |
|  | Oleoylcarnitine (18:1) | 4.73 | 1.51 | 2 | 2 ↑ | Nieman, 2015 (2) | - |  |
|  | Linoleoylcarnitine (18:2) | 4.32 | 1.98 | 2 | 2 ↑ | Nieman, 2015 (2) | - |  |
|  | Arachidonoylcarnitine (20:4) | 0.39 | 0.00 | 1 | 1 ↑ | Prado, 2017 | 1 ↓ | Howe, 2018 |
|  | Isovalerylcarnitine | 1.60 | 1.34 | 2 | 2 ↑ | Nieman, 2013; Prado, 2017 | 1 ↓ | Nieman, 2013 |
|  | Dehydroxycarnitine | 2.33 | 0.62 | 2 | 2 ↑ | Daskalaki, 2015 (2) | - |  |

**Table S2 continued.**

| **a)**  Pathway | **b)**  Metabolite | **c)**  In 31 experiments n changes were reported. | | | **d)**  In all 57 experiments n increases were reported | | **e)**  In all 57 experiments n decreases were reported | |
| --- | --- | --- | --- | --- | --- | --- | --- | --- |
|  |  | MFC | SD | n |  |  | MFC | SD |
| **Ketone Bodies** | 3-Hydroxybutyrate | 7.48 | 10.89 | 8 | 6 ↑ | Pechlivanis, 2015; Karl, 2017; Nieman, 2015 (3); Peake, 2014 | 2 ↓ | Mukherjee, 2014 (2) |
|  | Acetoacetate | 6.03 | 8.46 | 5 | 3 ↑ | Karl, 2017; Nieman, 2013 (2) | 2 ↓ | Valerio, 2017 (2) |
|  | 3-Hydroxyisobutyrate | 1.59 | 0.49 | 9 | 8 ↑ | Pechlivanis, 2015; Mukherjee, 2014 (2); Pechlivanis, 2010 (2); Berton, 2016 (2); Nieman, 2013 | 1 ↓ | Nieman, 2013 |
|  | 2-Hydroxybutyrate | 1.97 | 0.53 | 12 | 13 ↑ | Pechlivanis, 2015; Mukherjee, 2014 (2); Pechlivanis, 2010 (2); Berton, 2016 (2); Peake, 2014 (5); Coelho, 2016 | - |  |
|  | Hydroxypentanoate | 2.06 | 0.94 | 2 | 2 ↑ | Daskalaki, 2015; Howe, 2018 | - |  |
|  | 2-Oxoisocaproate | 1.61 | 0.34 | 8 | 8 ↑ | Pechlivanis, 2015; Mukherjee, 2014 (2); Pechlivanis, 2010 (2); Berton, 2016 (3) | 1 ↓ | Prado, 2017 |
|  | Acetate | 1.5 | 0.30 | 2 | 4 ↑ | Pechlivanis, 2015 (2); Samudrala, 2015; Enea, 2010 | 1 ↓ | Prado, 2017 |
|  | Acetone (2-Propanone) | - | - | - | - |  | 2 ↓ | Messier, 2017 (2) |
| **Catabolism of ketogenic amino acids** | 6-Acetamido-3-oxohexanoate | 2.77 | 2.04 | 2 | 2 ↑ | Mukherjee, 2014 (2) | 1 ↓ |  |
|  | 3-Methyl-2-oxovalerate | 1.57 | 0.45 | 5 | 5 ↑ | Pechlivanis, 2015; Mukherjee, 2014 (2) Pechlivanis, 2010 (2) | - |  |
|  | 2-Oxoisovalerate | 1.23 | 0.11 | 5 | 5 ↑ | Pechlivanis, 2015; Mukherjee, 2014 (2); Pechlivanis, 2010 (2) | - |  |
|  | 3-Hydroxy-3-methylglutarate | 1.65 | 0.00 | 1 | 1 ↑ | Karl, 2017 | 1 ↓ | Prado, 2017 |
|  | 2-Aminoadipate | 2.12 | 1.90 | 2 | 1 ↑ | Nieman, 2013 | 1 ↓ | Nieman, 2013 |
| **Ketogenic amino acids** | Isoleucine^*^ | 0.66 | 0.16 | 7 | 3 ↑ | Ra, 2014; Lewis, 2010 (2) | 9 ↓ | Pechlivanis, 2015; Berton, 2016; Howe, 2018; Peake, 2014 (4); Messier, 2017; Lewis, 2010 (2) |
|  | Leucine* | 0.65 | 0.13 | 6 | 4 ↑ | Ra, 2014; Lewis, 2010 (2); Sun, 2018 | 10 ↓ | Berton, 2016; Muhsen, 2016; Howe, 2018; Peake, 2014 (3); Prado, 2017; Messier, 2017; Lewis, 2010; Coelho, 2016 |
|  | Lysine* | 0.76 | 0.37 | 3 | 2 ↑ | Breit, 2015; Chorell, 2011 | 5 ↓ | Berton, 2016; Howe, 2018; Lewis, 2010; Coelho, 2016; Danaher, 2015 |

*****duplicates of Table S3 (Amino Acids and Peptides)

**Table S2 continued.**

| **a)**  Pathway | **b)**  Metabolite | **c)**  In 31 experiments n changes were reported. | | | **d)**  In all 57 experiments n increases were reported | | **e)**  In all 57 experiments n decreases were reported | |
| --- | --- | --- | --- | --- | --- | --- | --- | --- |
|  |  | MFC | SD | n |  |  | MFC | SD |
| **Glycero-phospho-lipids /Sphingo-lipids** | 1-Palmitoyl-GPE (16:0) | 0.55 | 0.11 | 3 | - | - | 3 ↓ | Karl, 2017; Nieman, 2013 (2) |
|  | 1-Oleoyl-GPE (18:1) | 0.52 | 0.14 | 3 | - | - | 3 ↓ | Karl, 2017; Nieman, 2013 (2) |
|  | 2-Linoleoyl-GPE (18:2) | 0.32 | 0.10 | 2 | - | - | 2 ↓ | Nieman, 2013 (2) |
|  | 1-Arachidonoyl-GPE (20:4n6) | 0.62 | 0.24 | 3 | - |  | 3 ↓ | Karl, 2017; Nieman, 2013 (2) |
|  | Sphingosine-1-phosphate | 0.81 | - | 1 | 1 ↑ | Prado, 2017 | 1 ↓ | Karl, 2017 |
| **Steroids** | 11b-Hydroxyandrost-4-ene-3,17-dione | 3.93 | 2.28 | 2 | 2 ↑ | Nieman, 2015 (2) | - |  |
|  | Andro steroid monosulfate | 1.66 | - | 1 | 1 ↑ | Karl, 2017 | 1 ↓ | Prado, 2017 |
|  | Cortisone | 1.35 | 0.61 | 2 | 1 ↑ | Howe, 2018 | 1 ↓ | Karl, 2017 |
|  | Etiocholanolone glucuronide | 0.91 | - | 1 | 1 ↑ | Prado, 2017 | 1 ↓ | Karl, 2017 |
|  | Androstenediol (3alpha, 17alpha) monsulfate | 0.57 | - | 1 | - |  | 2 ↓ | Karl, 2017; Prado, 2017 |
|  | Cholestane-tetrol-glucuronide | 0.58 | 0.12 | 3 | - |  | 3 ↓ | Mukherjee, 2014 (2); Howe, 2018 |
| **Bile Acids** | Cholate | 0.86 | 1.48 | 4 | - |  | 4 ↓ | Karl, 2017; Muhsen, 2016; Nieman, 2013 (2) |
|  | Glycocholate | 0.63 | 1.18 | 3 | - |  | 3 ↓ | Howe, 2018; Nieman, 2013 (2) |
|  | Taurocholate | 0.69 | 1.16 | 3 | - |  | 3 ↓ | Howe, 2018; Nieman, 2013 (2) |
|  | Glycochenodeoxycholate | 0.52 | 0.80 | 2 | - |  | 2 ↓ | Nieman, 2013 (2) |
|  | Glycochenodeoxycholate glucuronide | 0.60 | 0.47 | 2 | 1 ↑ | Prado, 2017 | 1 ↓ | Karl, 2017 |
|  | Deoxycholate | 0.30 | 0.42 | 1 | - |  | 1 ↓ | Karl, 2017 |
|  | Glycodeoxycholate | 0.49 | 0.81 | 2 | - |  | 2 ↓ | Nieman, 2013 (2) |
|  | Taurodeoxycholate | 0.54 | 0.79 | 2 | - |  | 2 ↓ | Nieman, 2013 (2) |
|  | Taurolithocholate 3-sulfate | 1.89 | - | 1 | 2 ↑ | Karl, 2017; Prado, 2017 | - |  |

**Table S3.** **Amino Acids and Peptides**

| **a)**  Pathway | **b)**  Metabolite | **c)**  In 31 experiments n changes were reported. | | | **d)**  In all 57 experiments n increases were reported | | **e)**  In all 57 experiments n decreases were reported | |
| --- | --- | --- | --- | --- | --- | --- | --- | --- |
|  |  | MFC | SD | n |  |  | MFC | SD |
| **Glucogenic amino acids** | Alanine | 1.30 | 0.45 | 14 | 24 ↑ | Breit, 2015; Pechlivanis, 2015 (BL); Mukherjee, 2014 (2) Pechlivanis, 2010 (2); Berton, 2016 (3); Valerio, 2017; Peake, 2014; Ra, 2014; Zauber, 2012; Enea, 2010; Lewis, 2010 (4); Chorell, 2011 (2); Danaher, 2015 (2), Zafeiridis, 2016; Sun, 2018 | 4 ↓ | Howe, 2018; Peake, 2014 (2); Messier, 2017 |
|  | Asparagine | 0.63 | - | 1 | 1 ↑ | Chorell, 2011 | 5 ↓ | Hooton, 2016; Prado, 2017; Lewis, 2010; Danaher, 2015 (2) |
|  | Aspartate | 0.88 | - | 1 |  |  | 1 ↓ | Breit, 2015 |
|  | Arginine | 1.36 | 0.00 | 1 | 1 ↑ | Breit, 2015 | 3 ↓ | Coelho, 2016 (2); Lewis, 2010 |
|  | Glutamate | 1.04 | 0.48 | 3 | 3 ↑ | Breit, 2015; Peake, 2014; Zauber, 2012 | 3 ↓ | Howe, 2018; Coelho, 2016; Danaher, 2015 |
|  | Glutamine | 0.71 | - | 1 | 5 ↑ | Zauber, 2012; Lewis, 2010 (4) | 5 ↓ | Howe, 2018; Messier, 2017; Lewis, 2010; Coelho, 2016; Zafeiridis, 2016 |
|  | Glycine | 0.78 | 0.17 | 9 | 4 ↑ | Breit, 2015; Prado, 2017; Ra, 2014; Hall, 2016 | 8 ↓ | Pechlivanis, 2015 (2); Mukherjee, 2014 (2); Pechlivanis, 2010 (2); Howe, 2018; Sun, 2018 |
|  | Histidine | 0.74 | 0.31 | 4 | 1 ↑ | Breit, 2015 | 4 ↓ | Daskalaki, 2015; Pechlivanis, 2010 (2); Lewis, 2010 |
|  | Methionine | 0.93 | 0.68 | 7 | 6 ↑ | Daskalaki, 2015; Breit, 2015; Prado, 2017; Lewis, 2010 (2); Coelho | 5 ↓ | Muhsen, 2016; Howe, 2018; Peake, 2014 (3) |
|  | Proline | 1.33 | 1.46 | 5 | 3 ↑ | Daskalaki, 2015; Breit, 2015; Lewis, 2010 | 5 ↓ | Howe, 2018; Peake, 2014 (2); Zauber, 2012; Zafeiridis, 2016 |
|  | Serine | 0.81 | 0.42 | 2 | 1 ↑ | Breit, 2015 | 5 ↓ | Howe, 2018; Lewis, 2010 (4) |
|  | Valine | 0.86 | 0.18 | 10 | 3 ↑ | Breit, 2015; Mukherjee, 2014; Ra, 2014 | 11 ↓ | Pechlivanis, 2015 (2); Mukherjee, 2014; Berton, 2016; Howe, 2018; Peake, 2014 (3); Prado, 2017; Messier, 2017; Lewis, 2010 |
| **Glucgenic and ketogenic amino acids** | Isoleucine | 0.66 | 0.16 | 7 | 3 ↑ | Ra, 2014; Lewis, 2010 (2) | 9 ↓ | Pechlivanis, 2015; Berton, 2016; Howe, 2018; Peake, 2014 (4); Messier, 2017; Lewis, 2010 (2) |
|  | Phenylalanine | 0.94 | 0.12 | 4 | 2 ↑ | Daskalaki, 2015; Ra, 2014 | 3 ↓ | Karl, 2017; Mukherjee, 2014; Peake, 2014 |
|  | Threonine | 0.65 | 0.44 | 3 | 2 ↑ | Breit, 2015; Chorell, 2011 | 4 ↓ | Muhsen, 2016; Howe, 2018; Chorell, 2011; Lewis, 2010 |
|  | Tryptophan | 1.02 | 0.47 | 7 | 4 ↑ | Daskalaki, 2015; Breit, 2015; Ra, 2014; Chorell, 2011 | 8 ↓ | Mukherjee, 2014 (2); Pechlivanis, 2010 (2); Howe, 2018; Prado, 2017; Chorell, 2012; Lewis, 2010 |
|  | Tyrosine | 0.91 | 0.23 | 5 | 2 ↑ | Breit, 2015; Peake, 2014 | 3 ↓ | Pechlivanis, 2015; Howe, 2018; Peake, 2014 |
| **Ketogenic amino acids** | Leucine | 0.65 | 0.13 | 6 | 4 ↑ | Ra, 2014; Lewis, 2010 (2); Sun, 2018 | 10 ↓ | Berton, 2016; Muhsen, 2016; Howe, 2018; Peake, 2014 (3); Prado, 2017; Messier, 2017; Lewis, 2010; Coelho, 2016 |
|  | Lysine | 0.76 | 0.37 | 3 | 2 ↑ | Breit, 2015; Chorell, 2011 | 5 ↓ | Berton, 2016; Howe, 2018; Lewis, 2010; Coelho, 2016; Danaher, 2015 |
| **Other amino acids** | Citruline | 0.90 | 0.32 | 2 | 1 ↑ | Breit, 2015 | 4 ↓ | Howe, 2018; Lewis, 2010 (3) |
|  | Ornithine | 0.81 | 0.34 | 3 | 4 ↑ | Breit, 2015; Zauber, 2012; Chorell, 2012; Coelho, 2016 | 5 ↓ | Berton, 2016; Howe, 2018; Lewis, 2010 (3) |
|  | Beta-alanine | 0.84 | - | 1 | 1 ↑ | Chorell, 2011 | 1 ↓ | Karl, 2017 |
|  | O-Acetyl-L-homoserine | 2.41 | 1.69 | 3 | 2 ↑ | Daskalaki, 2015 (2) | 1 ↓ | Howe, 2018 |
|  | 3-Hydroxytryptophan | 1.94 | 1.08 | 2 | 2 ↑ | Daskalaki, 2015; Karl, 2017 | 1 ↓ | Prado, 2017 |
|  | Phenylacetylglycine | 1.76 | 0.00 | 1 | 2 ↑ | Coelho, 2016; Prado, 2017 | - |  |
|  | Trimethylamin n-oxide | 0.83 | 0.04 | 6 | - |  | 6 ↓ | Pechlivanis, 2015 (2); Mukherjee, 2014 (2); Pechlivanis, 2010 (2) |
|  | Glutarate | - | - | - | 1 ↑ | Zauber, 2012 | 1 ↓ | Prado, 2017 |
|  | Cysteine | - | - | - | 1 ↑ | Prado, 2017 | 2 ↓ | Lewis, 2010 (2) |
|  | Cystine | - | - | - | - |  | 1 ↓ | Prado, 2017 |
| **Biogenic amines** | Creatine | 0.54 | - | 1 | 2 ↑ | Lewis, 2010; Coelho, 2016 | 2 ↓ | Prado, 2017; Muhsen, 2016 |
|  | Creatinine | 0.60 | 0.20 | 3 | 4 ↑ | Prado, 2017; Zauber, 2012; Lewis, 2010; Sun, 2018 | 3 ↓ | Peake, 2014 (2); Pechlivanis, (2015) |
|  | Histamine | 0.50 | - | 1 | 1 ↑ | Prado, 2017 | 1 ↓ | Hooton, 2016 |
|  | Kynurenate | 2.32 | 0.00 | 1 | 2 ↑ | Howe, 2018; Lewis, 2010 | - |  |
|  | Taurine | 0.57 | 0.00 | 1 | 3 ↑ | Ra, 2014; Chorell, 2011; Coelho, 2016 | 3 ↓ | Howe, 2018; Prado, 2017; Sun, 2018 |
|  | Aniline | 5.37 | 0.65 | 2 | 2 ↑ | Nieman, 2015 (2) | - |  |
|  | Urocanate | 1.31 | 0.97 | 2 | 1 ↑ | Daskalaki, 2015 | 1 ↓ | Howe, 2018 |
|  | Betaine | 0.51 | - | 1 | - |  | 2 ↓ | Howe, 2018; Lewis, 2010 |
|  | Choline | 0.92 | 0.04 | 3 | - | - | 3 ↓ | Karl, 2017; Valerio, 2017 (2) |
| **Amino acid metabolism** | Imidazoleacetate | 0.61 | - | 1 | - | - | 2 ↓ | Hooton, 2016; Prado, 2017 |
|  | N-acetylphenylalanine | 1.54 | 1.39 | 2 | 1 ↑ | Nieman, 2013 | 1 ↓ | Nieman, 2013 |
|  | 5-Hydroxyindolepyruvate | 3.68 | 1.64 | 3 | 3 ↑ | Daskalaki, 2015 (2); Howe, 2018 | - |  |
|  | 4-Hydroxyphenylpyruvate | 2.53 | 1.6 | 3 | 3 ↑ | Daskalaki, 2015; Lewis, 2010, Nieman, 2013 | 1 ↓ | Nieman, 2013 |
|  | Xanthurenate | 3.25 | 1.91 | 2 | 2 ↑ | Daskalaki, 2015 (2) | - |  |
|  | Hydroxyphenyllactate | 0.54 | 0.00 | 1 | - |  | 2 ↓ | Howe, 2018; Prado, 2017 |
|  |  |  |  |  |  |  |  |  |
|  | Hexanoylglycine | 6.61 | 0.05 | 2 | 2 ↑ | Nieman, 2015 (2) | - |  |
|  | 4-Aminohippurate | 0.63 | - | 1 | - |  | 2 ↓ | Pechlivanis, 2015; Prado, 2017 |
|  | 3-Phenyllactic acid | - | - | - | 1 ↑ | Zauber, 2012 | 1 ↓ | Prado, 2017 |
| **Catecholamine** | L-Metanephrine | 3.30 | 1.84 | 2 | 2 ↑ | Daskalaki, 2015 (2) | - |  |
|  | N-Acetylvanilalanine | 5.35 | 3.04 | 2 | 2 ↑ | Daskalaki, 2015 (2) | - |  |
| **Urea cycle metabolites** | N-(Carboxyethyl) arginine | 4.30 | 1.27 | 2 | 2 ↑ | Daskalaki, 2015 (2) | - |  |
|  | Argininosuccinate | 0.61 | 0.21 | 2 | 1 ↑ | Lewis, 2010 | 2 ↓ | Muhsen, 2016; Howe, 2018 |
|  | Pyrrole-2-carboxylate | 0.41 | 0.00 | 1 | 1 ↑ | Prado, 2017 | 1 ↓ | Danaher, 2015 |
| **Peptides** | Cystathione | - | - | - | - | - | 2 ↓ | Lewis, 2010 (2) |
|  | Glycylproline | 0.69 | 0.02 | 3 | - | - | 3 ↓ | Hooton, 2016; Karl, 2017; Danaher, 2015 |
|  | Glycyl-L-leucine | 0.6 | 0.06 | 2 | - | - | 3 ↓ | Hooton, 2016; Danaher, 2015; Prado, 2017 |

**Table S4.** **Nucleotides**

| **a)**  Pathway | **b)**  Metabolite | **c)**  In 31 experiments n changes were reported. | | | **d)**  In all 57 experiments n increases were reported | | **e)**  In all 57 experiments n decreases were reported | |
| --- | --- | --- | --- | --- | --- | --- | --- | --- |
|  |  | MFC | SD | n |  |  | MFC | SD |
| **Purine Metabolism** | Inosine | 69.62 | 191.84 | 9 | 14 ↑ | Daskalaki, 2015 (2); Pechlivanis, 2015 (3); Karl, 2017; Mukherjee, 2014 (2); Lewis, 2010 (4); Muhsen, 2016; Sun, 2017 | - |  |
|  | Deoxyinosine | 21.29 | 19.79 | 3 | 3 ↑ | Daskalaki, 2015 (2); Muhsen, 2016 | - |  |
|  | Hypoxanthine | 9.77 | 7.24 | 13 | 22 ↑ | Daskalaki, 2015 (2); Prado, 2017; Pechlivanis, 2015 (3); Mukherjee, 2014 (2); Pechlivanis, 2010 (2); Berton, 2016 (2); Chorell, 2011; Enea, 2010; Lewis, 2010 (5); Muhsen, 2016; Howe, 2018; Sun, 2017 | - |  |
|  | Xanthosine | 8.92 | 6.95 | 3 | 6 ↑ | Daskalaki, 2015 (2); Lewis 2010 (3); Muhsen, 2016 | 1 ↓ | Prado, 2017 |
|  | N2-N2-Dimethylguanosine | 1.84 | 1.06 | 2 | 2 ↑ | Daskalaki, 2015; Karl, 2017 | - |  |
|  | Urate | 1.02 | 0.38 | 2 | 3 ↑ | Karl, 2017; Lewis, 2010; Coelho, 2016 | 1 ↓ | Muhsen, 2016 |
|  | N6-methyladenosine | 0.72 | - | 1 | 1 ↑ | Prado, 2017 | 1 ↓ | Karl, 2017 |
|  | Adenosine-5-monophosphate (AMP) | - | - | - | 5 ↑ | Chorell, 2011 (2), Lewis, 2010 (2) | - |  |
|  | Xanthine | - | - | - | 5 ↑ | Lewis, 2010 (5) | - |  |
|  | Allantoin | - | - | - | - | - | 4 ↓ | Lewis, 2010 (4) |
| **Pyrimidine Metabolism** | Guanine | 3.91 | 1.25 | 3 | 4 ↑ | Daskalaki, 2015 (2); Prado, 2017; Muhsen, 2016 | - |  |
|  | Cytidine | 1.32 | - | 1 | 2 ↑ | Prado, 2017; Karl, 2017 | - |  |
|  | Cytidine triphosphate | 1.09 | 0.08 | 2 | 2 ↑ | Mukherjee, 2014 (2) | 1 ↓ | Prado, 2017 |
|  | Uridine | 0.42 | - | 1 | 2 ↑ | Lewis, 2010 (2) | 2 ↓ | Howe, 2018; Prado, 2017 |

**Table S5.** **Cofactors/Vitamins and Xenometabolites**

| **a)**  Pathway | **b)**  Metabolite | **c)**  In 31 experiments n changes were reported. | | | **d)**  In all 57 experiments n increases were reported | | **e)**  In all 57 experiments n decreases were reported | |
| --- | --- | --- | --- | --- | --- | --- | --- | --- |
|  |  | MFC | SD | n |  |  | MFC | SD |
| **Cofactors or Vitamins** | Pantothenate | 3.22 | 1.85 | 3 | 8 ↑ | Daskalaki, 2015 (2); Muhsen, 2016; Lewis, 2010 (5) | - | - |
|  | Riboflavin | 1.84 | 1.66 | 2 | 2 ↑ | Daskalaki, 2015; Prado, 2017 | 1 ↓ | Muhsen, 2016 |
|  | Alpha-CECH | 1.01 | 0.04 | 2 | 1 ↑ | Mukherjee, 2014 | 2 ↓ | Mukherjee, 2014; Prado, 2017 |
|  | Niacinamide | 0.77 | 0.61 | 5 | 1 ↑ | Lewis, 2010 | 4 ↓ | Lewis, 2010 (4) |
|  | Gamma-Tocopherol | 0.53 | - | 1 | - | - | 2 ↓ | Howe, 2018; Chorell, 2011 |
|  | Threonate | - | - | - | 1 ↑ | Chorell, 2011 | 1 ↓ | Chorell, 2011 |
| **Xenometabolites** | Benzoate | 1.06 | 0.40 | 2 | 2 ↑ | Zauber, 2012; Peake, 2014 | 2 ↓ | Peake, 2014; Prado, 2017 |
|  | Hippurate | 0.83 | 0.09 | 5 | 1 ↑ | Lehmann, 2010; | 7 ↓ | Pechlivanis, 2015 (3); Mukherjee, 2014 (2); Lewis 2010 (2) |
|  | Gluconate | 0.76 | - | 1 | 2 ↑ | Zauber, 2012 (2) | 1 ↓ | Karl, 2017 |
|  | Acesulfame | 0.68 | 0.52 | 3 | 1 ↑ | Mukherjee, 2014 | 2 ↓ | Karl, 2017; Mukherjee, 2014 |
|  | Quinate | 0.32 | - | 1 | 2 ↑ | Zauber, 2012 (2) | 1 ↓ | Karl, 2017 |
