# Supplementary material for: Metabolite Concentration Changes in Humans After a Bout of Exercise: a Systematic Review of Exercise Metabolomics Studies
Source: Sports Med Open. 2020 Feb 10;6:11. doi: 10.1186/s40798-020-0238-4 (PMC7010904; doi:10.1186/s40798-020-0238-4)
Supplement: Supplementary file 2 — Additional file 2: Table S6. Descriptive summary of 57 experiments that reported metabolites concentration changes after a bout of exercise. [file 40798_2020_238_MOESM2_ESM.docx]

**Supplementary Table S6**

**Table S6. Descriptive summary of 57 experiments that reported metabolites concentration changes after a bout of exercise.**

**c)** Subject categories are separated into endurance trained subjects (End), Untrained subjects (Untr.) and others (not endurance trained). **h)**Tissues analyzed are categorized into plasma (PLA), serum (SER), urine (URI), saliva (SAL), sweat (SUD) and capillary blood (CAP). **n)** Intensity of endurance exercise intervention are categorized into: max=maximum exercise test; self-paced= no information on intensity given, IAT=individual anaerobic threshold (65-80% VO_2max_), aerobic (<65% VO_2max_) and supramax (>100% of VO_2max_, typically interval exercise). The intensities are given as mean intensity of the protocol. Intensities of resistance exercise are quantified in % of 1RPM (one repetition maximum weight). **e), f)** and **g)** are reported as means ± standard deviation. **o)** Duration of exercise intervention is reported as the actual time of exercise without break times. If a protocol consisted of different exercise intensities, the entire duration of the exercise protocol is given. “-“ no information was provided on this parameter

| a)  Author, Year | **b)**  N | **c)**  Subjects | **d)**  sex | **e)**  Endurance capacity  VO_2max_ | **f)**  Age | **g)**  Bodyfat | **h)**  Tissue analyzed | **i)**  Analysis technique | **j)**  Sampling time | | | **k)**  Fasting^[[1]](#footnote-1)^ | **m)**  Standard diet^[[2]](#footnote-2)^ | **n)**  Intensity of exercise intervention | **o)**  Duration of exercise intervention |
| --- | --- | --- | --- | --- | --- | --- | --- | --- | --- | --- | --- | --- | --- | --- | --- |
|  |  |  | (♂, ♀) | (ml/kg/min) | (years) | (in %) |  |  | BL=  baseline  sample | 1^st^ post-exercise sample (hours) | 2^nd^ post-exercise sample (hours) |  |  |  | (minutes) |
| (Lewis et al., 2010) | 45 | Other | 8♀, 37♂ | - | 58 ±13 | - | PLA | LC-MS | BL | 0 | 1 h | no | - | Max | 30^3^ |
|  | 25 | other | 2♀, 23♂ | - | 59 ±12 | - | PLA |  | BL | 0 | 1 h | no | - | Max | 30^3^ |
|  | 25 | End. | 6♀, 19♂ | - | 42 ±9 | - | PLA |  | BL | 10 | - | - | - | self-paced | 180-300^3^ |
| (Enea et al., 2010) | 10 | Untr. | ♀ | 33.8 ±1.8 | 22.1 ±0.6 | 23.7 ±0.8 | URI | HNMR | BL | 0.5 | - | no | yes | IAT | 26.7 ± 1.8 |
|  | 6 | End. | ♀ | 55.8 ±1.3 | 23.0 ±1.9 | 20.0 ±0.8 | URI |  | BL | 0.5 | - | no | yes | IAT | 52.7 ± 3 |
|  | 6 | other | ♀ | 41.5 ±1.2 | 20.7 ±0.7 | 22.9 ±1.5 | URI |  | BL | 0.5 | - | no | yes | IAT | 29.5 ± 2.4 |
| (Pechlivanis, 2010 #349) | 12 | other | ♂ | - | 20 ±1,  21 ±2 | 22.0 ±1.5 | URI | HNMR | BL | 0.583 | - | no | yes | Max | 0.167^[[3]](#footnote-3)^ |
| (Lehmann et al., 2010) | 13 | - | ♂ | 56.5 ±1.4 | 32.6 ±6.1 | - | PLA | LC-MS | BL | 0 | 3, 24 | yes | no | IAT | 60 |
|  | 8 | - | ♂ | 63 ±2 | 30.9 ±5.8 | - | PLA |  | BL | 0 | 4, 24 | yes | no | aerobic | 120 |
| (Chorell et al., 2012) | 6 | End. | ♂ | 63.7 ±2.8 | 25.6 ±1.8 | 12.8 ±2.3 | PLA | GC-MS | BL | 0 | - | yes | yes | IAT | 65 |
|  | 7 | Untr. | ♂ | 42.7 ±2.9 | 24 ±1.8 | 24.5 ±4 | PLA |  | BL | 0 | - | yes | yes | IAT | 65 |
| (Zauber et al., 2012) | 2 | End. | ♀1, 1♂ | - | - | - | SAL | GC-MS/ LC-MS | ^[[4]](#footnote-4)^ | 6 | - | - | no^[[5]](#footnote-5)^ | self-paced | 3720 |
| (Nieman et al., 2013) | 15 | End. | ♀8, ♂7 | ♂ 56.9 ±5.8  ♀ 51.9 ±5.9 | - | 16.3 ±4.7  22.7 ±9.1 | SER | LC-MS/UHPLC- MS/MS | BL | 0 | 14 | - | no | IAT | 150  (for 3 consecutive days) |
| (Peake et al., 2014) | 10 | End. | ♂ | - | 33.2 ±6.7 | - | SER, PLA | GC-MS | BL | 0 | 1, 2 | - | no | IAT | 60 |
| (Mukherjee et al., 2014) | 9 | End. | ♂ | 59.1 ±5.2 | 53.4 ±3.2 | - | URI | HNMR | BL | 24 | - | yes | no | anaerobic | 45 |
|  | 8 | Untr. | ♂ | 35.9 ±9.7 | 54.3 ±5.0 | - | URI |  | ^3^ | 24 | - | yes | no | anaerobic | 46 |
| (Ra et al., 2014) | 37 | End. | ♂ | - | 20.6 ±0.04 | - | SAL | CE-TOF-MS | BL | 12 | - | yes^[[6]](#footnote-6)^ | - | self-paced | 90 |
| (Danaher et al., 2015) | 7 | End. | ♂ | 50.0 ±6.1 | 22.9 ±5.0 | - | PLA | GC-MS | BL | 0 | 1 | yes | yes | Supramax | 15 |
| (Nieman et al., 2015) | 20 | End. | ♂ | 51.0 ±1.4 | 39.2 ±1.9 | 17.7 ±1.1 | Blood, other | LC-MS/MS | BL | 0 | 1.5, 21 | no^2^ | - | self-paced | 120-180^3^ |
| (Daskalaki et al., 2015) | 3 | End. | ♂ | - | 32–38 | - | URI | LC-MS | BL | 1 | - | no | - | self-paced | ~50 |
| (Pechlivanis et al., 2015) | 17 | other | ♂ | - | 19 ±1 | - | URI | RP-UPLC-MS & HNMR | - | 1 | 1.5 | no | - | Max | 0.167^3^ |
| (Breit et al., 2015) | 47 | End. | ♀20, ♂27 | - | 34.2 | - | CAP (dried) | MS-MS | BL | 0.083 | - | - | - | Max (Testing) | 51 |
| (Samudrala et al., 2015) | 23 | - | ♀10, ♂13 | - | 56 ±13 | - | URI | PTR-MS | BL | 0 | - | - | - | self-paced | 2880 |
| (Muhsen Ali et al., 2016) | 10 | Untr. | ♀2, ♂8 | 42.1±5.4 | 28 ±7 | - | URI | LC-MS | BL | 1 | 24 | no | - | aerobic | 45 |
| (Coelho et al., 2016) | 4 | End. | ♂ | - | - | 11.5 ±2.0 | SER, PLA | HPLC | BL | 0 |  | -^2^ | no | self-paced | 210 |
| (Berton et al., 2017) | 10 | other | ♂ | - | 24 ±2 |  | SER | HNMR | BL | 0.083 | 0.25, 0.5, 1 | no | no | 70% 1-RPM (RE) | 0.5^3^ |
| (Hall et al., 2016) | 13 | End. | ♀4, ♂9 | ♀ 62 ±2  ♂68 ±1 | 32 ±2 |  | SER, PLA | NMR | BL | 0 | - | no | yes | IAT | 60 |
| (Hooton et al., 2016) | 6 | - | ♀3, ♂3 | - | - | - | SUD | LC-MS | ^[[7]](#footnote-7)^ | 0.5-0.67 | - |  |  | self-paced | 40 |
| (Zafeiridis et al., 2016) | 9 | End. | ♂ | 59.7 ±1.2 | 20.5 ±0.7 | - | PLA | HNMR | BL | 0.083 | - | no | yes | IAT | 64.3 ± 4.7 |
| (Prado et al., 2017) | 30 | End. | ♂ |  | 19±1 | - | URI | LC-MS | BL | 0 | - |  |  | self-paced | 90 |
| (Messier et al., 2017) | 20 | - | ♂ | 53 ±8 | 39 ±4.3 | 18.9 ±3.6 | PLA | HNMR | BL | 0 | - | no | yes | IAT | 60 |
| (Karl et al., 2017) | 25 | other | ♂ | - | 19 ±1 | - | PLA | LC-MS | BL | 2-3 or 8-9 | | yes | yes | self-paced | 2880 |
| (Valerio et al., 2017) | 9 | Res. | ♂ | - | 26.4 ±4.4 | - | SER | HNMR | BL | 0.083 | - | no | no | self-paced (RE) | 3 |
| (Sun et al., 2017) | 19 | End. | ♂ | - | - | - | URI | NMR | BL | - | - | - | - | -- | - |
| (Howe et al., 2018) | 9 | End. | ♂ | 61.6 ±4.3 | 34 ±7 | - | PLA | LC-MS | BL | 0 | - | no^1^ | no | IAT | 557 ± 78 |

**References** (also included in manuscript)

Berton, R., Conceicao, M.S., Libardi, C.A., Canevarolo, R.R., Gaspari, A.F., Chacon-Mikahil, M.P.T., Zeri, A.C., and Cavaglieri, C.R. (2017). Metabolic time-course response after resistance exercise: A metabolomics approach. Journal of sports sciences *35*, 1211-1218.

Breit, M., Netzer, M., Weinberger, K.M., and Baumgartner, C. (2015). Modeling and Classification of Kinetic Patterns of Dynamic Metabolic Biomarkers in Physical Activity. Plos Computational Biology *11*.

Chorell, E., Svensson, M.B., Moritz, T., and Antti, H. (2012). Physical fitness level is reflected by alterations in the human plasma metabolome. Mol Biosyst *8*, 1187-1196.

Coelho, W.S., Viveiros de Castro, L., Deane, E., Magno-Franca, A., Bassini, A., and Cameron, L.C. (2016). Investigating the Cellular and Metabolic Responses of World-Class Canoeists Training: A Sportomics Approach. Nutrients *8*.

Danaher, J., Gerber, T., Wellard, R.M., Stathis, C.G., and Cooke, M.B. (2015). The use of metabolomics to monitor simultaneous changes in metabolic variables following supramaximal low volume high intensity exercise. Metabolomics *12*.

Daskalaki, E., Blackburn, G., Kalna, G., Zhang, T., Anthony, N., and Watson, D.G. (2015). A Study of the effects of exercise on the urinary metabolome using normalisation to individual metabolic output. Metabolites *5*, 119-139.

Enea, C., Seguin, F., Petitpas-Mulliez, J., Boildieu, N., Boisseau, N., Delpech, N., Diaz, V., Eugene, M., and Dugue, B. (2010). (1)H NMR-based metabolomics approach for exploring urinary metabolome modifications after acute and chronic physical exercise. Anal Bioanal Chem *396*, 1167-1176.

Hall, U.A., Edin, F., Pedersen, A., and Madsen, K. (2016). Whole-body fat oxidation increases more by prior exercise than overnight fasting in elite endurance athletes. Applied Physiology, Nutrition and Metabolism *41*, 430-437.

Hooton, K., Han, W., and Li, L. (2016). Comprehensive and Quantitative Profiling of the Human Sweat Submetabolome Using High-Performance Chemical Isotope Labeling LC-MS. Analytical chemistry *88*, 7378-7386.

Howe, C.C.F., Alshehri, A., Muggeridge, D., Mullen, A.B., Boyd, M., Spendiff, O., Moir, H.J., and Watson, D.G. (2018). Untargeted Metabolomics Profiling of an 80.5 km Simulated Treadmill Ultramarathon. Metabolites *8*.

Karl, J.P., Margolis, L.M., Murphy, N.E., Carrigan, C.T., Castellani, J.W., Madslien, E.H., Teien, H.K., Martini, S., Montain, S.J., and Pasiakos, S.M. (2017). Military training elicits marked increases in plasma metabolomic signatures of energy metabolism, lipolysis, fatty acid oxidation, and ketogenesis. Physiol Rep *5*.

Krug, S., Kastenmuller, G., Stuckler, F., Rist, M.J., Skurk, T., Sailer, M., Raffler, J., Romisch-Margl, W., Adamski, J., Prehn, C.*, et al.* (2012). The dynamic range of the human metabolome revealed by challenges. FASEB J *26*, 2607-2619.

Lehmann, R., Zhao, X., Weigert, C., Simon, P., Fehrenbach, E., Fritsche, J., Machann, J., Schick, F., Wang, J., Hoene, M.*, et al.* (2010). Medium chain acylcarnitines dominate the metabolite pattern in humans under moderate intensity exercise and support lipid oxidation. PLoS ONE *5*.

Lewis, G.D., Farrell, L., Wood, M.J., Martinovic, M., Arany, Z., Rowe, G.C., Souza, A., Cheng, S., McCabe, E.L., Yang, E.*, et al.* (2010). Metabolic signatures of exercise in human plasma. Sci Transl Med *2*, 33ra37.

Messier, F.M., Le Moyec, L., Santi, C., Gaston, A.F., Triba, M.N., Roca, E., and Durand, F. (2017). The impact of moderate altitude on exercise metabolism in recreational sportsmen: a nuclear magnetic resonance metabolomic approach. Applied Physiology Nutrition and Metabolism *42*, 1135-1141.

Muhsen Ali, A., Burleigh, M., Daskalaki, E., Zhang, T., Easton, C., and Watson, D.G. (2016). Metabolomic Profiling of Submaximal Exercise at a Standardised Relative Intensity in Healthy Adults. In Metabolites.

Mukherjee, K., Edgett, B.A., Burrows, H.W., Castro, C., Griffin, J.L., Schwertani, A.G., Gurd, B.J., and Funk, C.D. (2014). Whole blood transcriptomics and urinary metabolomics to define adaptive biochemical pathways of high-intensity exercise in 50-60 year old masters athletes. PLoS One *9*, e92031.

Nieman, D.C., Gillitt, N.D., Sha, W., Meaney, M.P., John, C., Pappan, K.L., and Kinchen, J.M. (2015). Metabolomics-Based Analysis of Banana and Pear Ingestion on Exercise Performance and Recovery. J Proteome Res *14*, 5367-5377.

Nieman, D.C., Shanely, R.A., Gillitt, N.D., Pappan, K.L., and Lila, M.A. (2013). Serum metabolic signatures induced by a three-day intensified exercise period persist after 14 h of recovery in runners. J Proteome Res *12*, 4577-4584.

Peake, J.M., Tan, S.J., Markworth, J.F., Broadbent, J.A., Skinner, T.L., and Cameron-Smith, D. (2014). Metabolic and hormonal responses to isoenergetic high-intensity interval exercise and continuous moderate-intensity exercise. Am J Physiol Endocrinol Metab *307*, E539-552.

Pechlivanis, A., Papaioannou, K.G., Tsalis, G., Saraslanidis, P., Mougios, V., and Theodoridis, G.A. (2015). Monitoring the response of the human urinary metabolome to brief maximal exercise by a combination of RP-UPLC-MS and 1H NMR spectroscopy. Journal of Proteome Research *14*, 4610-4622.

Prado, E., Souza, G.H.M.F., Pegurier, M., Vieira, C., Lima-Neto, A.B.M., Assis, M., Guedes, M.I.F., Koblitz, M.G.B., Ferreira, M.S.L., Macedo, A.F.*, et al.* (2017). Non-targeted sportomics analyses by mass spectrometry to understand exercise-induced metabolic stress in soccer players. International Journal of Mass Spectrometry *418*, 1-5.

Ra, S.G., Maeda, S., Higashino, R., Imai, T., and Miyakawa, S. (2014). Metabolomics of salivary fatigue markers in soccer players after consecutive games. Applied Physiology, Nutrition and Metabolism *39*, 1120-1126.

Samudrala, D., Geurts, B., Brown, P.A., Szymańska, E., Mandon, J., Jansen, J., Buydens, L., Harren, F.J.M., and Cristescu, S.M. (2015). Changes in urine headspace composition as an effect of strenuous walking. Metabolomics *11*, 1656-1666.

Sun, T., Wu, Y., Wu, X., and Ma, H. (2017). Metabolomic profiles investigation on athletes' urine 35 minutes after an 800-meter race. Journal of Sports Medicine and Physical Fitness *57*, 839-849.

Tsuda, M., Kinugawa, S., Fukushima, A., Matsushima, S., Furihata, T., Takada, S., Kadoguchi, T., Kudo, M., Matsumoto, J., Yokota, T.*, et al.* (2014). Changes of Metabolomic Profiling Are Associated With Reduced Exercise Capacity in Patients With Heart Failure. Circulation *130*.

Valerio, D.F., Berton, R., Conceicao, M.S., Canevarolo, R.R., Chacon-Mikahil, M.P.T., Cavaglieri, C.R., Meirelles, G.V., Zeri, A.C., and Libardi, C.A. (2017). Early metabolic response after resistance exercise with blood flow restriction in well-trained men: a metabolomics approach. Appl Physiol Nutr Metab.

Zafeiridis, A., Chatziioannou, A.C., Sarivasiliou, H., Kyparos, A., Nikolaidis, M.G., Vrabas, I.S., Pechlivanis, A., Zoumpoulakis, P., Baskakis, C., Dipla, K.*, et al.* (2016). Global Metabolic Stress of Isoeffort Continuous and High Intensity Interval Aerobic Exercise: A Comparative 1H NMR Metabonomic Study. J Proteome Res *15*, 4452-4463.

Zauber, H., Mosler, S., von Hessberg, A., and Schulze, W.X. (2012). Dynamics of salivary proteins and metabolites during extreme endurance sports – a case study. Proteomics *12*, 2221-2235.

1. for at least 8 hours before the exercise [↑](#footnote-ref-1)
2. for at least 1 day before the trial day [↑](#footnote-ref-2)
3. duration estimated from the exercise protocol [↑](#footnote-ref-3)
4. pre-exercise sample was taken 24-48 h before the exercise intervention [↑](#footnote-ref-4)
5. non-standardized food was allowed during exercise [↑](#footnote-ref-5)
6. Additional standardized food was allowed during exercise [↑](#footnote-ref-6)
7. pre-exercise sample was taken 10-20 min into exercise. [↑](#footnote-ref-7)
