# Supplementary material for: Metabolite Concentration Changes in Humans After a Bout of Exercise: a Systematic Review of Exercise Metabolomics Studies
Source: Sports Med Open. 2020 Feb 10;6:11. doi: 10.1186/s40798-020-0238-4 (PMC7010904; doi:10.1186/s40798-020-0238-4)
Supplement: Supplementary file 3 — Additional file 3: Table S7. Metabolites changed in relation to time point of sampling (with Fig. 4). [file 40798_2020_238_MOESM3_ESM.pdf]

Supplementary Table S7 (with Figure 4). Metabolites changed in relation to time point of sampling

Changed at all timepoints (31 metabolites)	Changed in early and intermediate sampling experiments (38 metabolites)	Changed in early and late sampling experiments (10 metabolites)
**Acylcarnitines**<br>Hexanoylcarnitine (6:0)<br>Octanoylcarnitine (8:0)<br>Decanoylcarnitine (10:0)<br>Decenoylcarnitine (10:1)<br>Dodecanoylcarnitine (12:0)<br>**Fatty acids**<br>Tetradecanoate (14:0)<br>9-Tetradecenoate (14:1n5)<br>Hexadecenoate (16:1n7)<br>Octadecenoate (18:1)<br>Linoleate (18:2)<br>Linolenate (18:3n3)<br>Octadecatetraenoate (18:4n3)<br>Eicosenoate (20:1)<br>Dihomo-linoleate (20:2n6)<br>Dihomo-linolenate (20:3n3 or n6)<br>Eicosapentaenoate (20:5n3)<br>Docosadienoate (22:2n6)<br>Docosapentaenoate (22:5n3)<br>Docosahexaenoate (22:6n3)<br>Dodecanedioate (12:0)<br>Tetradecanedioate (14:0)<br>Hexadecanedioate (16:0)<br>Heptadecanoate (17:0)<br>10-Heptadecenoate (17:1n7)<br>10-Nonadecenoate (19:1n9)<br>**Ketone bodies**<br>3-Hydroxybutyrate<br>Acetoacetate<br>**Glycerophospholipids**<br>1-Palmitoyl-GPE (16:0)<br>1-Oleoyl-GPE (18:1)<br>1-Arachidonoyl-GPE (20:4n6)<br>**Bile acids**<br>Cholate	**Acylcarnitines**<br>Tetradecanoylcarnitine(14:0)<br>Myristoleoylcarnitine (14:1)<br>Oleoylcarnitine (18:1)<br>Linoleoylcarnitine (18:2)<br>**Fatty acids**<br>Decanedioate (10:0)<br>Dodecanoate (12:0)<br>5-Dodecenoate (12:1n7)<br>Pentadecanoate (15:0)<br>Linoleate (18:2) a or g<br>Docosatrienoate (22:3)<br>9-Heptadecanoate (17:0)<br>3-Hydroxydecanoate<br>3-Hydroxydodecanoate<br>3-Hydroxydecanedioate<br>Hexanoylglycine<br>**Ketone bodies**<br>2-Hydroxybutyrate<br>**TCA cycle intermediates**<br>Itaconate<br>Malate<br>Aconitate<br>**Steroids**<br>11b-Hydroxyandrost-4-ene-3,17-dione<br>Cortisone<br>**Amino acids**<br>2-Aminoadipate<br>3-Aminoisobutyrate<br>Aniline<br>Alanine<br>Choline<br>Isoleucine<br>Leucine<br>Proline<br>Lysine<br>Methionine<br>Ornithine<br>Phenylalanine<br>Tyrosine<br>Valine<br>**Carbohydrate metabolism**<br>Glycerol<br>**Vitamins & Cofactors**<br>Niacinamide<br>Xenobiotics<br>Benzoate	**Acylcarnitines**<br>Isovalerylcarnitine<br>**Fatty acids**<br>Octadecanedioate (18:0)<br>**Glycerophospholipids**<br>2-Linoleoyl-GPE<br>**Amino acids**<br>4-Hydroxyphenylpyruvate<br>N-acetylphenylalanine<br>**Bile acids**<br>Glycochenodeoxycholate<br>Glycocholate<br>Glycodeoxycholate<br>Taurocholate<br>Taurodeoxycholate

Changed in early sampling experiments only (44 metabolites)	Changed in intermediate sampling experiments only (26 metabolites)	Changed in late sampling experiments only (1 metabolites)
**Acylcarnitines**<br>Acetylcarnitine (2:0)<br>Propionylcarnitine (3:0)<br>Butyrylcarnitine (4:0)<br>Pentanoylcarnitine (5:0)<br>Octenoylcarnitine (8:1)<br>Nonanoylcarnitine (9:0)<br>Dodecenoylcarnitine (12:1)<br>Tetradecanoylcarnitine (14:0)<br>Tetradecenoylcarnitine/isomer (14:1)<br>Arachidonoylcarnitine (20:<br>6-Keto-decanoylcarnitine<br>**Amino acids**<br>Arginine<br>Arginosuccinate<br>Aspartate<br>Betaine<br>Citrulline<br>Glutamate<br>Glutamine<br>Glycine<br>Histidine<br>Kynurenate<br>Pyrrole-2-carboxylate<br>O-acetyl-l-homoserine<br>Serine<br>Threonine<br>Tryptophan<br>5-Hydroxyindolepyruvate<br>Hydroxyphenyllactate<br>Taurine<br>Uridine<br>Urocanate<br>**Fatty acids**<br>Hydroxypentanoate<br>Hexadecanedioate monocarnitineester<br>**Carbohydrate metabolism**<br>Glucose<br>Lactate<br>Rhamnose or isomer<br>**TCA cycle intermediates**<br>Citrate<br>Succinate<br>Pyruvate<br>**Nucleotides**<br>Hypoxanthine<br>**Vitamins and Cofactors**<br>Gamma-tocopherol<br>**Ketone bodies**<br>2-Oxoisocaproate<br>**Steroids**<br>Cholestane-tetrol-glucuronide	**Fatty acids**<br>Octanoate (8:0)<br>Octadecanoate (18:0)<br>Hexadecanoate (16:0)<br>Docosanoate (22:0)<br>**Nucleotides**<br>Cytidine<br>Inosine<br>Urate<br>N2-N2-Dimethylguanosine<br>N6-Methyladenosine<br>**Amino acid & peptides**<br>3-Hydroxy-3-methylglutarate<br>3-Hydroxytetradecanedioate<br>3-Hydroxytryptophan<br>Beta-alanine<br>Creatinine<br>Glycyl-L-leucine<br>**Carbohydrate metabolism**<br>Myo-inositol<br>**Xenobiotics**<br>Acesulfame<br>Erythritol<br>Quinate<br>Gluconate<br>**Glycerophospholipids**<br>Sphingosine-1-phosphate<br>**Bile acids**<br>Glycochenodeoxycholate glucuronide<br>Taurolithocholate-3-sulfate<br>**Steroids**<br>Androsteroid monosulfate<br>Androstenediol (3a, 17a) monosulfate<br>Etiocholanolone glucuronide	**Ketone bodies**<br>3-Hydroxyisobutyrate
